# DBF4 Dependent Kinase Inhibition Suppresses Hepatocellular Carcinoma Progression and Potentiates Anti-Programmed Cell Death-1 Therapy

**DOI:** 10.7150/ijbs.80351

**Published:** 2023-07-03

**Authors:** Liang Zhang, Jiawei Hong, Wei Chen, Wei Zhang, Xi Liu, Jiahua Lu, Hong Tang, Zhentao Yang, Ke Zhou, Haiyang Xie, Changku Jia, Donghai Jiang, Shusen Zheng

**Affiliations:** 1 Division of Hepatobiliary and Pancreatic Surgery, Department of Surgery, The First Affiliated Hospital, Zhejiang University School of Medicine, Hangzhou, China.; 2 Key Laboratory of Organ Transplantation, NHC Key Laboratory of Combined Multi-organ Transplantation, Hangzhou, China.; 3 Department of Hepatobiliary and Pancreatic Surgery, Affiliated Hangzhou First People's Hospital, Zhejiang University School of Medicine, Research Center of Diagnosis and Treatment Technology for Hepatocellular Carcinoma of Zhejiang Province, Hangzhou, China.; 4 Department of Medical Oncology, Sir Runrun Shaw Hospital, College of Medicine, Zhejiang University, Hangzhou, China.

**Keywords:** DDK, STAT3, XPO1, liver cancer, combined therapy

## Abstract

The progression of hepatocellular carcinoma (HCC) remains a huge clinical challenge, and elucidation of the underlying molecular mechanisms is critical to develop effective therapeutic strategy. Dumbbell former 4 (DBF4) complexes with cell division cycle 7 (CDC7) to form DBF4-dependent kinase (DDK), playing instrumental roles in tumor cell survival, whereas its roles in HCC remain elusive. This study revealed that DBF4 expression was upregulated in HCC and constituted an independent prognostic factor of patient survival. We identified p65 as an upstream inducer which increased DBF4 expression by directly binding to its promoter. DBF4 accelerated HCC cell proliferation and tumorigenesis* in vitro* and *in vivo*. Mechanistically, DBF4 complexed with CDC7 to bind to the coiled coil domain of STAT3 and activate STAT3 signaling through XPO1-mediated nuclear exportation. Notably, p65 enhanced the nuclear transport of DDK and DDK-STAT3 interaction by transcriptionally upregulating XPO1. DBF4 expression positively correlated with activated STAT3 and XPO1 in HCC tissues. Furthermore, combining DDK inhibitor XL413 with anti-PD-1 immunotherapy dramatically suppressed HCC growth and prolonged the survival of HCC-bearing mouse. Our findings reveal that DDK activates STAT3 pathway and facilitates HCC progression, and demonstrate the proof of the concept of targeting DDK to improve the efficacy of HCC immunotherapy.

## Background

Hepatocellular carcinoma (HCC) is a common and highly aggressive tumor that has been recognized as the seventh most common malignancy and the fourth leading cause of cancer-related death globally.^1^, ^2^ Although the recent decades have witnessed the substantial progress in the clinical management of HCC, including curative resection, interventional therapy, liver transplantation to targeted therapy or immunotherapy, the estimated 5-year survival rate of HCC remains as low as 14-18%, largely owing to its insidious and aggressive nature.^3^ Therefore, efforts are underway to further improve our understanding on the molecular pathogenesis of HCC aiming to develop novel therapeutic strategies and reduce mortality of this malignancy.

Proper nuclear-cytoplasmic partitioning of specific molecules is critical in maintaining cellular homeostasis, which is frequently dysregulated during tumorgenesis. Smaller molecules (<40kDa) are known to shuttle between the nucleus and cytoplasm via simple diffusion, while the transport of larger proteins and certain RNAs requires specialized carriers.^4^ A total of seven exportins have been so far identified to mediate the export of nuclear biomolecules.^5^ Among which, exportin 1 (XPO1) as the most widely characterized nuclear exporter is the only exportin responsible for the transport of proteins containing nuclear export signal (NES).^6^ XPO1 is frequently overexpressed in various types of human cancers, which induces the mislocalization and consequent dysfunction of tumour suppressor proteins and cell cycle mediators, functioning as a critical oncogenic driver. While it was previously reported that XPO1 was aberrantly elevated in HCC and promoted tumor cell growth,^7^ the mechanisms underlying the function and regulation of XPO1 in HCC remain to be fully elucidated.

Aberrancy in cell cycle progression is a major feature of human cancers. DBF4-dependent kinase (DDK), an evolutionarily conserved complex composed of dumbbell former 4 (DBF4) and cell division cycle 7 (CDC7), play critical roles in DNA synthesis, chromosomal segregation, DNA damage response and replication stress.^8^ In addition, DDK was found to be instrumental for the survival of cancer cells.^9^ Pharmacological inhibition of DDK induces tumor-specific cell death while showing preserved cell viability in non-transformed cells.^10^ DDK thus holds considerable value as an attractive therapeutic target for cancer treatment. DBF4 is the critical regulatory subunit of DDK which controls the functional activity of CDC7 by facilitating the ATP binding and substrate recognition of CDC7. 11, 12 DBF4 was reported to be expressed at low levels in normal tissues while aberrantly upregulated in multiple types of cancers.^13^, ^14^ For example, studies were reported that DBF4 as an molecular determinant with prognostic relevance was increased in melanoma cells and conferred a proliferative advantage.^15^ DBF4 was also upregulated in lung cancer and promoted tumor growth and invasiveness.^16^ In addition, elevated expression of DBF4 was associated with gastric cancer progression, aggressiveness, and resistance to 5‑Fu chemotherapy.^17^ Nevertheless, the biological role and underlying mechanisms of DBF4 in the regulation of HCC progression have not yet been explored.

In this study, we demonstrate that DBF4, as a pro-oncogenic factor, is upregulated in HCC and independently correlated with inferior patient survival. Suppressing or increasing DBF4 expression attenuates or enhances HCC cell proliferation, respectively. DBF4 binds to and activates STAT3 via complexing with CDC7, which involves the XPO1-mediated nuclear transportation. Furthermore, our data shows that pharmacological inhibition of DDK function may be a valuable therapeutic approach for the treatment of HCC.

## Methods

### Clinical specimen collection

Clinical samples were obtained from 107 patients with primary HCC who underwent curative hepatic resection between 2013 and 2016 at the First Affiliated Hospital, College of Medicine, Zhejiang University. Data on patient demographics and clinicopathological characteristics, including age (≤60 years or >60 years), gender (male or female), hepatitis B virus infection (with or without), microvascular invasion (present or absent), cirrhosis (present or absent), serum alpha-fetoprotein level (≤400 ng/mL or >400 ng/mL), number of nodules (solitary or multiple), maximum tumor diameter (≤5cm or >5cm) and histopathological differentiation (grading I-II or III-IV, according to Edmondson-Steiner criteria) were collected and utilized in this study (Table 1). Patient survival was calculated from the date of operation to death or censored at last follow up.

### Cell culture

The human HCC cell lines HCCLM3, MHCC97H, Huh7 and SNU449 were purchased from Shanghai Institutes of Biological Sciences. Mouse HCC cell line Hepa1-6 was obtained from American Type Culture Collection. HEK293T cells and an immortalized human hepatocyte HepLi5 were kind gifts from Prof. Lanjuan Li (First Affiliated Hospital, Zhejiang University School of Medicine, Hangzhou, China).^18^-^20^ A normal mouse liver cell line, alpha mouse liver 12 (AML-12), was purchased from Procell Life Science & Technology Co.,Ltd. Cells were cultured in Dulbecco's Modified Eagle's Medium and maintained in a humidified incubator with 5% CO_2_ at 37°C. The medium was supplied with 10% fetal bovine serum and 1% penicillin-streptomycin. All cell lines were cultured within 20 passages after thawing and tested to be free of mycoplasma contamination.

### Immunohistochemistry (IHC) and immunofluorescence (IF)

The IHC and IF staining were performed as previously described.^21^, ^22^ In brief, the tissue sections were immersed in 3% H_2_O_2_ solution after deparaffinization. Antigen retrieval was performed in a heated 10 mmol/L citrate buffer solution (pH 6.0) or 1 mmol/L EDTA solution (pH 9.0) as appropriate. After cooling down to room temperature, the sections were washed with phosphate buffered saline (PBS), blocked with fetal bovine serum, and thereafter incubated with the primary antibodies overnight at 4°C. The antibodies used in this study included DBF4 (Santa Cruz, #sc-293398), p-STAT3 (Tyr705) (CST, #9145), CD8 (CST, #98941), CD44 (ABclonal, #A1351), PD-1 (Abcam, #214421), Granzyme B (CST, #44153), XPO1 (ABclonal, #A19625), and Ki-67 (Abcam, #ab16667).

For IHC, the sections were incubated with the anti-mice/rabbit enzyme-labeled secondary antibodies. Afterwards, the sections were treated with diaminobenzidine, rinsed in water, and eventually counterstained with haematoxylin. Negative controls to show the specificity of the immunostaining included omission of the primary antibody incubation step and substitution with the primary antibody diluent (Figure S1). Staining performance was qualified using 3D Histech Quantcenter 2.1 software, and the extent of immunoreactivity was assessed by H-score, which captures both the staining intensity and area. For IF, the secondary antibodies used in our study were the anti-mouse IgG antibodies gated with Alexa Fluor 594 (CST, #4408S), and/or anti-rabbit IgG antibodies gated with Alexa Fluor 594 (CST, #8889S). 4,6-diamidino-2-dole (DAPI) was finally used to counterstain nuclei.

### Stable transfection using lentiviral infection

Lentiviruses expressing sh-DBF4 or sh-NC, Flag-DBF4 (OE-DBF4) or empty vector (OE-NC) were purchased from Genechem (Shanghai, China). To generate stable DBF4 depletion or overexpression HCC cells, 2×10^5^ cells were seeded into a 6-well plate and transfected with the indicated lentivirus according to the manufacturers' instructions. Transfected cells were selected with 5 μg/mL puromycin (MCE, USA) for more than three weeks, and the transfection efficiency was examined by RT-qPCR and immunoblotting analysis. The shRNA target sequences are provided in Table S1.

### Small interfering RNA and plasmids

The siRNAs used in this study were synthesized by GenePharma (Shanghai, China), and the siRNA sequences are listed in Table S1. The plasmids were purchased from RiboBio (GuangZhou, China). Transfections of siRNAs and plasmids were performed using jetPRIME Polyplus Kit (France) according to the manufacturer's instructions. The cells were harvested 48 h after transfection for RT-qPCR examination or after 72 h for immunoblotting or further analysis.

### RNA purification purification, reverse transcription and RT-qPCR

Total RNA of the tissue and cells was extracted using FastPure Cell/Tissue Total RNA Isolation 167 Kit V2 (Vazyme Biotech Co.,Ltd, China) according to manufactures' instructions, and a Nano Drop 2000 system (Thermo Fisher, Carlsbad, USA) was used for the measurement of the RNA concentration. HiScript II Q RT SuperMix for qPCR (Vazyme Biotech Co.,Ltd, China) was used to synthesize cDNA, and subsequently, qRT-PCR was conducted using ChamQ Universal SYBR qPCR Master Mix (Vazyme Biotech Co., Ltd, China) on QuantStudio^TM^ 5 Real-Time PCR System (Thermo Fisher, USA). Quantification of relative messenger RNA (mRNA) expression was computed by the 2^ΔΔCt^ method after normalization to the GAPDH. The PCR primers were synthesized by Tsingke Biological Technology (Beijing, China) and provided in Table S2.

### Chromatin immunoprecipitation (ChIP) assay

ChIP assay in HEK293T cells was performed using the Simple ChIP^®^ Enzymatic Chromatin IP Kit (CST, #9003s) according to the manufacturer's instructions. Cells were harvested followed by crosslinking with 1% formaldehyde for 10 minutes. Chromatin DNA was digested with micrococcal nuclease for 20 min at 37°C to obtain fragments with lengths of 150 to 900 bp. The DNA was eluted and digested with proteinase K at 65 °C for 2 hours, and the purified DNA was analyzed by RT-qPCR. The primer sequences are provided in Table S2.

### Western blotting and co-immunoprecipitation assay

The tissue or cell proteins were extracted using the RIPA buffer supplemented with 1% Protease Inhibitor Cocktail (Thermo Fisher Scientific, USA), and the concentration of extracted proteins was measured by the BCA Protein Assay Kit (Pierce, USA). Protein denaturation was carried out by heating at 100°C for 15 minutes in 1× loading buffer (Invitrogen, USA). After separation by electrophoresis in 4-20% SDS-PAGE gels, the proteins were transferred to a 0.2μm polyvinylidene fluoride membrane (Millipore, USA). The membranes were then blocked with 5% skim milk at room temperature for 2 h, and subsequently incubated with their respective primary antibodies at 4°C overnight. The primary antibodies used in this study were listed in the Table S3. The membranes were washed with TBST and incubated with a horseradish peroxidase- conjugated secondary antibody at room temperature for 1 h. The signals were eventually detected with an FDbio-Femto ECL Kit (Hangzhou Fude Biotech Co.,Ltd, China).

For coimmunoprecipitation assay, the Dynabeads Co-Immunoprecipitation Kit (Thermo Fisher Scientific, USA) was used in accordance with the manufacturer's guidelines. Briefly, the beads were incubated with the corresponding antibodies or IgG on a roller at 37°C overnight. The conjugated beads were incubated with cell lysate on a rotator at 4°C for 30 minutes. Afterwards, the beads were washed and eluted with elution buffer.

### Cell nucleus/cytoplasm fraction isolation

For isolation of cytoplasmic and nuclear fraction, we employed a nuclear and cytoplasmic protein extraction kit (Beyotime Biotechnology, China). Pretreated cells were harvested and resuspended in buffer A for 15 min on ice. The cells were then centrifugated at 12000× g at 4 °C for 5 min after the addition of buffer B, and the supernatant was collected as the cytoplasmic fraction. The centrifuged pellet was repeatedly resuspended in nuclear protein extraction buffer for 30 min on ice. After centrifugation at 12000× g for 10 min, the supernatant obtained represents the nuclear fraction. Levels of GAPDH and Histone H3 were used as normalization controls for the cytoplasmic and nuclear fractions, respectively.

### Dual luciferase reporter assay

The sequences of the wild-type or mutant-type 3′ untranslated region sites of DBF4 or XPO1 were respectively synthesized and constructed into pmirGLO vector (Repobio, China), as previously described.^23^, ^24^ Cells were transfected with the corresponding reporter plasmids using Lipofectamine 2000 (Invitrogen, USA). The pRL-TK reporter construct was used for internal control. The cells were harvested 48 hours after the transfection, washed twice with PBS, and lysed in culture dishes containing lysis buffer. The lysates were centrifuged at 12,000 rpm for 2 min. The luciferase activity was measured using a Dual luciferase Reporter Assay Kit (Vazyme Biotech Co.,Ltd, China) following the manufacturer's instructions.

### Duo-link proximity ligation assay

In order to show the binding between CDC7 and STAT3, HCCLM3 cells were subjected to the Duo-link proximity ligation assay (Sigma-Aldrich, DUO92101) with the anti-CDC7 (Abcam, #ab229187) and anti-STAT3 (CST, #9139) antibodies, according to the protocols provided by the manufacturer. The fluorescent images were captured by a TCS SP8 X confocal microscope (Leica).

### Cell proliferation and colony formation assays

For cell proliferation assay, cells were seeded into 96-well plates at a density of 1000 cells per well. Cell Counting Kit-8 (CCK-8; MedChemExpress, USA) reagent was added into respective wells and incubated at 37 °C for 1 h. The absorbance value at 450 nm wavelength was measured by Varioskan Flash (Thermo Scientific, USA). For colony formation assay, 1000 cells were seeded in a 6-well plate and incubated for 10-14 days with minor adjustments being made depending on the cell line characteristics. Afterwards, the plates were fixed in 4% paraformaldehyde and stained with 0.1% crystal violet.

### Cell apoptosis assay and cell cycle analysis

To detect apoptotic cells, the pre-treated cells were harvested after trypsinization using EDTA-free trypsin, washed twice with PBS, and stained with the Annexin V-APC/7-AAD apoptosis kit (Multiscience, China) according to the manufacturer's instructions. The apoptotic cells were examined by flow cytometry (BD Bioscience, USA), and the data was analyzed with FlowJo X 10.0.7 software. For cell cycle analysis, the cells were harvested at about 80% confluency and fixed with cold 70% ethanol at -20°C overnight. After discarding the ethanol, the cells were washed with PBS, and incubated with DNA staining solution (Multiscience, China) for 30 minutes at room temperature. The DNA content were measured by flow cytometry, and the data was analyzed using ModFit software.

### Xenograft tumor model

All animal experiments were approved by the Ethics Committee for Laboratory Animals of the First Affiliated Hospital of Zhejiang University, and were conducted in accordance with the National Institutes of Health Guide for the Care and Use of Laboratory Animals.

To develop the nude mouse subcutaneous xenograft model, 4 × 10^6^ indicated HCCLM3 or MHCC97H cells in the logarithmic phase were resuspended in 100 μL PBS and subcutaneously injected to the flanks of the 8-week-old BALB/C nude mice. Tumor sizes were measured every 5 to 7 days. 4 weeks after injection, mice were sacrificed, and tumors were harvested and processed for further analysis. To establish orthotopic xenograft model in immunocompetent mice, a 25-μL mixture with 0.5 × 10^6^ indicated Hepa1-6 cells and Matrigel was injected into the left liver lobe of C57BL/6 mouse (male, 8 weeks old) through an 8-mm transverse incision in the epigastrium under general anesthesia. For subcutaneous implantation, 0.2 × 10^6^ Hepa1-6 cells were subcutaneously implanted into right flank of mice. For drug-based interventions, XL413 (Selleck) was reconstituted in water (warmed with 50ºC water bath) and administered orally at 50 mg/kg, 6 days per week to treat mice. For antibody-based drug intervention, anti-PD-1 antibody (200 μg; Bio X Cell) or IgG (Bio X Cell) was injected i.p. every 3 days. The tumor weight was recorded and the tumor volume was calculated using the modified ellipsoidal formula (length × width^2^)/2.

### Cell treatment

Cells were seeded in 6-well plates or 10-cm dish and allowed to attach overnight. Afterwards, cells were treated with IL-6, TNF-α (Peprotech), XL413 (Selleck), caffeic acid phenethyl ester, or selinexor (MedChem Express) according to the methods described in the text.

### Statistical analysis

Continuous variables were described as the mean ± standard deviation and compared using Student's* t*-test or analysis of variance as appropriate. Categorical data were presented as the number (percentage), and Pearson's Chi-square test or Fisher's exact test were used for comparisons. Bivariate correlation analysis was conducted using Spearman's rank-order correlation. Survival analysis was computed by the Kaplan-Meier method and compared by the log rank test. Univariate and multivariate Cox survival analyses were performed to identify independent prognostic factors. A two-tailed *P* < 0.05 was considered statistically significant.

## Results

### DBF4 is elevated in HCC tissues and correlated with inferior prognosis

**Figure 1:**
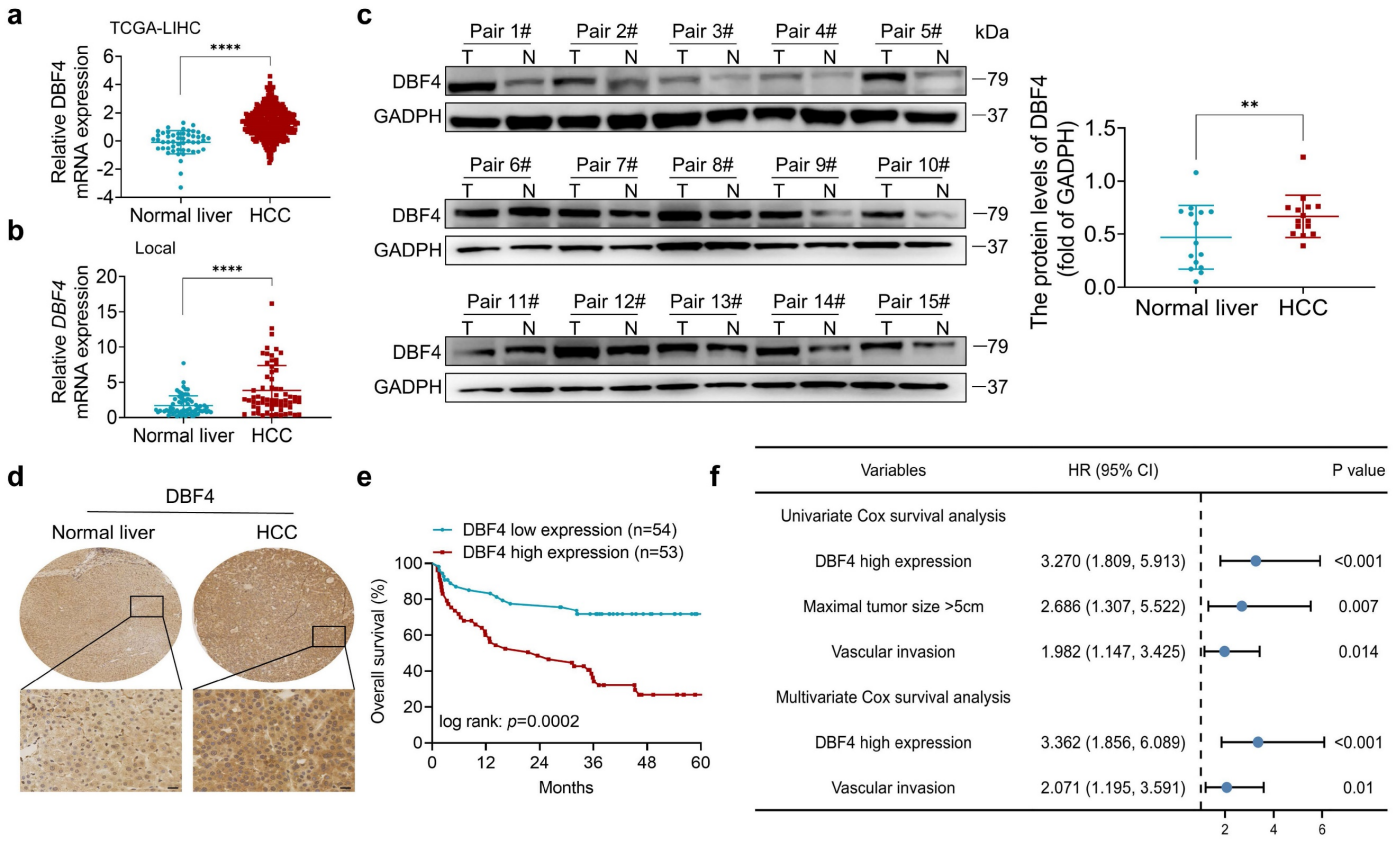
** DBF4 upregulation is correlated with poor prognosis in HCC patients.** (a). Bioinformatics analysis of DBF4 expression in HCC tissues according to the TCGA dataset. (b). RT-qPCR was employed to examine DBF4 mRNA expression in 66 paired HCC tissues and nontumoral liver tissues. (c). Western blotting was used to evaluate DBF4 abundance in 15 paired HCC tissues (T) and nontumoral tissues (N). GAPDH, glyceraldehyde-3-phosphate dehydrogenase. (d). Representative images of IHC staining of DBF4 in HCC tissues. Scale bars: 20 μm (400× magnification). (e). Kaplan-Meier survival curves stratified by DBF4 abundance based on IHC staining were generated for HCC patients (n = 107). (f). Univariate and multivariate Cox proportional hazards analyses were conducted to determine the hazard ratio (HR) of DBF4 expression for overall survival in HCC patients.

We initially analyzed the expression of DBF4 in HCC. According to The Cancer Genome Atlas (TCGA) dataset, we found that DBF4 expression was upregulated in HCC tissues (Figure 1a). Consistently, RT-qPCR assay of clinical samples from our cohort also suggested that HCC tissues had higher DBF4 mRNA level than paired nontumoral tissues (Figure 1b). Western blotting and IHC staining were used to detect protein expression in HCC tissues and adjacent tissues, which confirmed the upregulation of DBF4 expression in HCC tissues (Figure 1c-d). We noted that HCC patients with DBF4 high expression had larger tumor diameter (> 5cm; Table 1) and exhibited inferior survival (Figure 1e). Furthermore, univariate and multivariate analysis identified DBF4 high expression as an independent negative prognostic factor (Figure 1f). In addition, analysis of the TCGA dataset suggested that increased DBF4 expression was linked to advanced tumor T classification and shortened survival of HCC patients (Figure S2a-c). Collectively, these results suggest that DBF4 expression is elevated in HCC and has a potential clinical value for prognostic stratification in HCC patients.

### DBF4 accelerates HCC cell growth *in vitro* and *in vivo*

**Figure 2:**
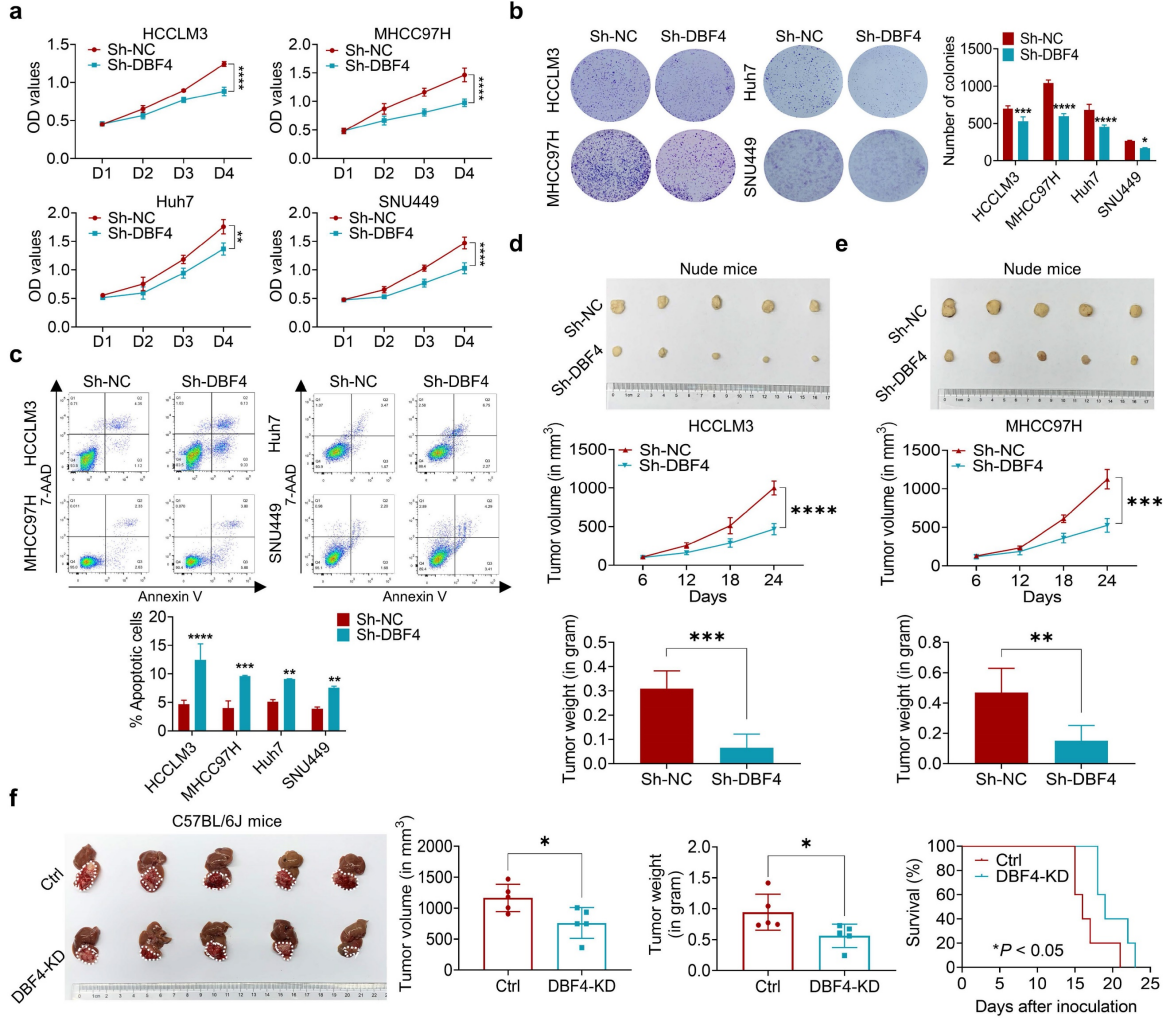
** DBF4 increases HCC cell proliferation* in vitro* and *in vivo*.** (a). CCK-8 assays showed the proliferative activities of HCC cells with DBF4 knockdown. (b). The effect of DBF4 knockdown on the colony forming capabilities of HCC cells. SNU449 cells showed less dense colonies. For this reason, the resultant purple dots that were relatively clustered together were counted as a colony. (c). Flow cytometric assays detecting the effects of DBF4 knockdown on apoptosis in HCC cells. (d, e). The HCC subcutaneous xenograft tumorigenesis models were established in immunodeficient nude mice using HCCLM3 and MHCC97H cells (for each group, n = 5). Macroscopic examination of tumor specimens (upper panel), tumor growth curves (middle panel), and qualifications of tumor weight (lower panel). (f). Hepa1‐6 cells with or without DBF4 knockdown (KD) were inoculated into the livers of C57BL/6 (for each group, n = 5). Tumor appearances (left), volume and weight (middle), and survival probability of mice (right) were displayed. * *P* <0.05, ** *P* < 0.01, ***P < 0.001, ****P < 0.0001.

Since DBF4 level was found to positively correlate with tumor size, we next set out to explore whether DBF4 affected HCC growth. CCK8 and colony formation assays showed that DBF4 depletion markedly impaired the proliferation of HCCLM3, MHCC97H, Huh7 and SNU449 cells (Figure 2a-b). Flow cytometry revealed that loss of DBF4 induced G1/S arrest and apoptosis of HCC cells (Figure S3 and Figure 2c). The opposite results were obtained after DBF4 overexpression in HCCLM3 and MHCC97H cells (Figure S4a-d), which further supported the oncogenic role of DBF4.

We next examined the effects of DBF4 on HCC tumorigenicity *in vivo*. DBF4- depleted and negative control HCCLM3 and MHCC97H cells were subcutaneously injected into nude mice to measure tumor growth. In line with our *in vivo* data, the volumes and weights of xenografted tumors in the knockdown group were reduced when compared with the control group (Figure 2d-e). IHC staining demonstrated that number of Ki-67 positive cells decreased in tumors derived from DBF4-depleted HCC cells (Figure S4e). Similarly, knockdown of DBF4 in Hepa1-6 cells repressed subcutaneous and orthotopic tumor growth, decreased tumoral Ki-67 expression, and extended the mouse survival (Figure 2f and Figure S4f-g). Therefore, these findings suggest DBF4 promotes HCC cell proliferation and tumorigenic ability *in vitro* and *in vivo*.

### DBF4 binds to and facilitates STAT3 activation

**Figure 3:**
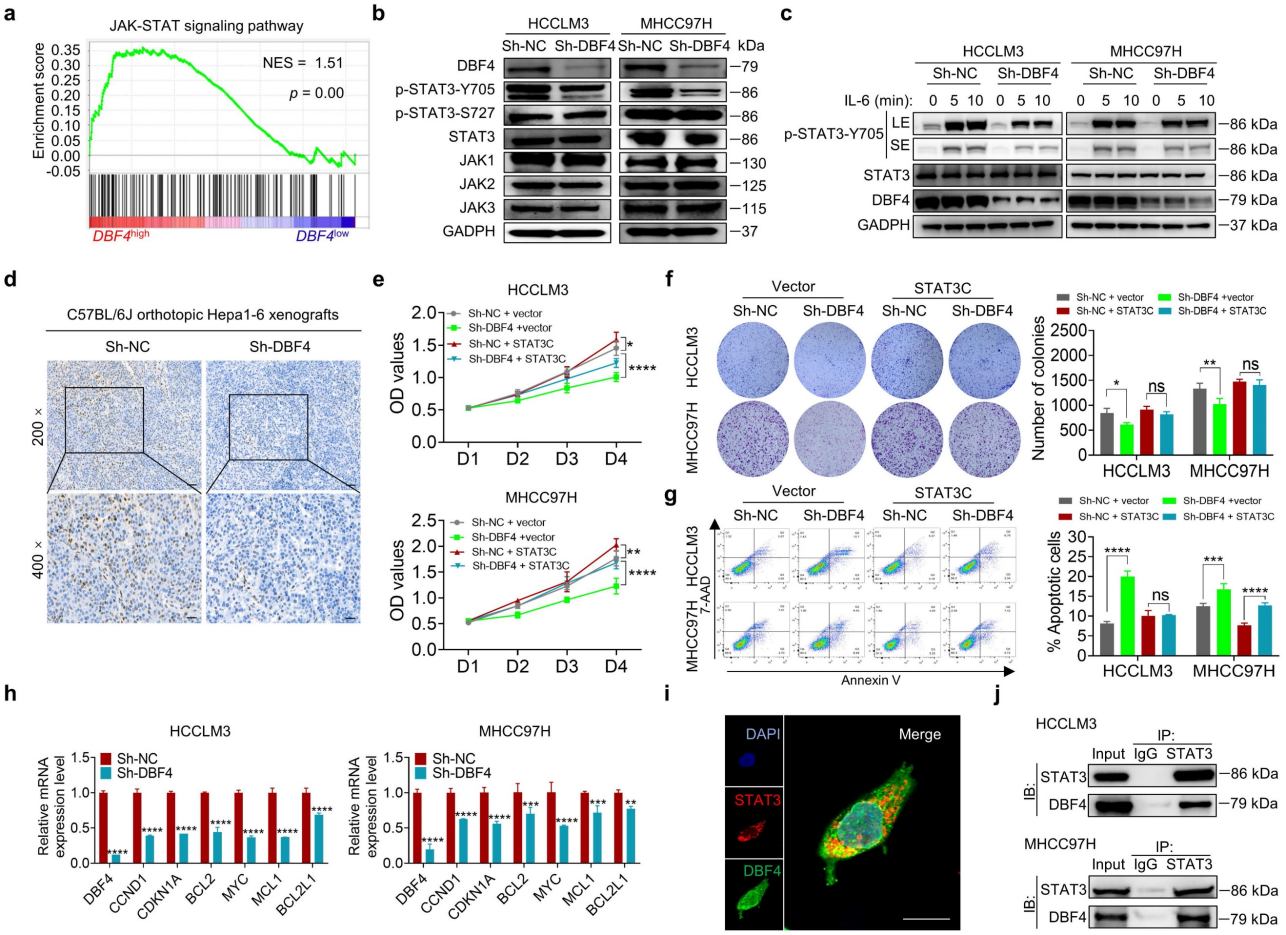
** DBF4 facilitates STAT3 signaling activation.** (a). Gene set enrichment analyses enrichment plot for the “JAK-STAT signaling” gene modules in the ranked gene list of with DBF4^high^ versus DBF4^low^ HCC samples from the GEO database (GSE76427). NES, normalized enrichment score. (b). The abundance of the indicated proteins was detected by immunoblotting in DBF4-depleted HCCLM3 or MHCC97H cells. (c). Western blot analysis showed the abundance of p-STAT3-Y705 in DBF4-depleted HCC cells treated with IL-6 (10 ng/mL) at the indicated time points. LE: longer exposure; SE: shorter exposure. (d). The expression of p-STAT3-Y705 was examined by IHC staining in DBF4-depelted Hepa1-6 orthotopic xenograft tumor tissues. Scale bars: 50 μm for 200× magnification and 20 μm for 400× magnification. (e-g). The proliferative activities (e), colony formation capabilities (f), and apoptosis (g) of DBF4-deplelted HCC cells transfected with STAT3C plasmids. (h). RT-qPCR assay was conducted to analyze several of STAT3 target genes mRNA levels in DBF4-depleted HCCLM3 and MHCC97H cells. (i). IF staining assay evaluating the colocalization between DBF4 and STAT3 in HCCLM3 cells. Scale bars: 10 μm. (j). The interaction between STAT3 and DBF4 in HCCLM3 and MHCC97H cells was evaluated by co-immunoprecipitation assays. IgG, immunoglobulin G; IB, immunoblotting; IP, immunoprecipitation. * *P* <0.05, ** *P* < 0.01, ***P < 0.001, ****P < 0.0001; ns, not significant.

To explore the signaling pathways affected by DBF4, we ranked 31425 genes from HCC samples in GEO database (GSE76427) by their relative DBF4 expression and used those in top quarter (DBF4^high^) and the bottom quarter (DBF4^low^) for gene set enrichment analyses (GSEAs; Figure S5a). DBF4^high^ HCC samples were significantly enriched in the expression of gene signatures associated with the JAK-STAT signaling pathway (Figure 3a). Our previous studies suggested that constitutive STAT3 activation plays an important role in promoting HCC progression.^25^, ^26^ We thus speculated whether DBF4 promoted HCC cell proliferation in a STAT3 pathway dependent manner. We evaluated the effect of DBF4 on JAK/STAT3 pathway and found that DBF4 depletion or overexpression inhibited or boosted STAT3 phosphorylation at Tyr 705 (p-STAT3-Y705) in HCC cells, respectively (Figure 3b and Figure S5b). Interleukin-6 (IL-6) is known to specifically activate STAT3 phosphorylation.^27^ IL-6 stimulation increased the abundance of p-STAT3-Y705 in HCCLM3 cells, while loss of DBF4 attenuated the activation efficiency of IL-6 (Figure 3c). In addition, analogous results could be verified in subcutaneous and orthotopic xenografts (Figure 3d and Figure S5c). Thus, these results indicate DBF4 regulates STAT3 activation in HCC cells.

To evaluate the magnitude of STAT3 activation in DBF4-mediated oncogenic phenotypes, we introduced a constitutively activated STAT3 mutant (STAT3C) in DBF4-depleted HCC cells. As the results showed, STAT3C rescued the growth defect and impaired viability of DBF4-depleted HCC cells to a great extent, indicating that the inhibitory effects were in part due to STAT3 inactivation induced by DBF4 depletion (Figure 3e-g). DBF4 was found to be positively correlated with STAT3 downstream target genes, including *CCND1*, *CDKN1A*, *MYC*, *MCL*, *BCL2* and *BCL2L1* (Figure S5d). We verified that DBF4 depletion decreased the expression of these genes in HCC cells (Figure 3h). In addition, coimmunoprecipitation and colocalization assays revealed DBF4 interacted with STAT3 in the cytoplasm (Figure 3i-j). These results suggest that DBF4 facilitates HCC progression by activating STAT3 pathway.

### DBF4 regulates STAT3 activity by complexing with CDC7

**Figure 4:**
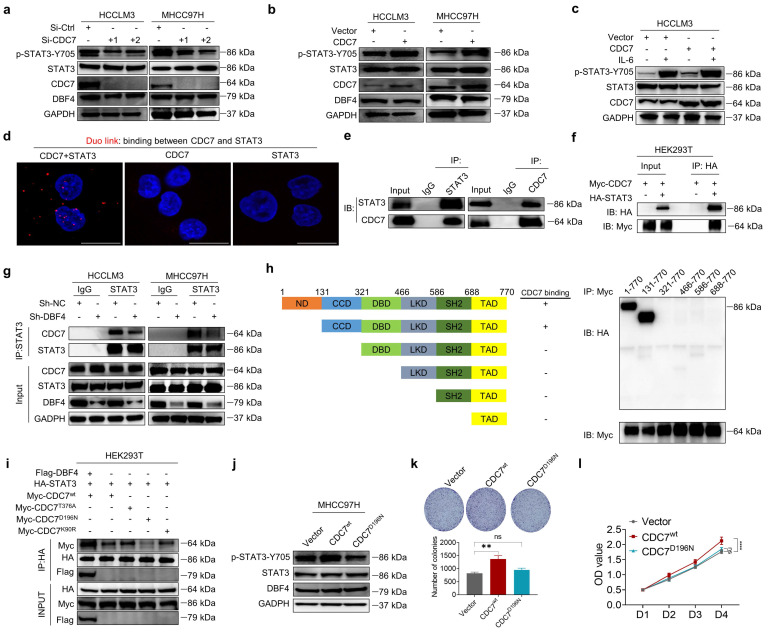
** CDC7 binds to and activates STAT3.** (a). The abundance of the indicated proteins was measured by western blotting in HCCLM3 and MHCC97H cells transfected with siRNAs targeting CDC7. (b). The expression of p-STAT3-Y705 was detected by western blotting in CDC7-overexpressing HCC cells. (c). CDC7-overexpressing HCCLM3 cells were treated with IL-6 (10 ng/mL) for 10 minutes, followed by western blotting analysis. (d). HCCLM3 cells were immunostained with anti-CDC7 and anti-STAT3 antibodies and assessed using Duolink proximity ligation assay. Red foci indicate the endogenous interaction between CDC7 and STAT3. Scale bars: 20 μm. (e). Reciprocal coimmunoprecipitation of CDC7 with STAT3 in HCCLM3. The immunoprecipitated materials by the indicated antibodies were subjected to western blotting analysis. (f). HEK293T cells were transfected with Myc tagged-CDC7 and/or HA tagged -STAT3. Whole cell lysates were immunoprecipitated with anti-HA antibody, with IgG as the negative control, and then the precipitates were analyzed with western blotting with anti-Myc antibody and anti-HA antibody. (g). Immunoprecipitation with an anti-STAT3 antibody or IgG was performed in HCC cells with or without the depletion of DBF4. (h). STAT3 consists of six structural domains, which was used for serial deletion analysis. HEK293T cells were cotransfected with truncated HA-tagged STAT3 and Myc-tagged CDC7. CDC7 was immunoprecipitated with anti-Myc antibody. Coimmunoprecipitated truncated STAT3 was then detected by anti-HA antibody. (i). Co-immunoprecipitation assays were performed in HEK293T cells transfected with the indicated expressing plasmids. (j). Immunoblot analysis was performed with the indicated antibodies in MHCC97H cells transfected with Myc-tagged wild type CDC7 (CDC7^wt^) or CDC7^D196N^. (k, l). Colony formation (k) and CCK-8 assays (l) of MHCC97H cells transfected with Myc- tagged CDC7^wt^ or CDC7^D196N^. ** *P* < 0.01, ****P < 0.0001; ns, not significant.

Now that DBF4 functions to complex with and modulate CDC7 activity,^28^ we examined whether DBF4 activated STAT3 pathway through CDC7. We found that, similar to the depletion of DBF4, siRNA-mediated silencing of CDC7 also inhibited the phosphorylation of STAT3 and its downstream target gene expression in HCC cells (Figure 4a and Figure S6a). Besides, ectopic CDC7 expression increased STAT3 phosphorylation (Figure 4b). The same effects were also found in the presence of IL-6 stimulation (Figure 4c). Furthermore, silencing of CDC7 completely abolished DBF4-mediated STAT3 activation, establishing a role of CDC7 in mediating STAT3 activity (Figure S6b).

Duolink proximity ligation assay and coimmunoprecipitation assay revealed that CDC7 could directly interact with STAT3 in HCCLM3 cells (Figure 4d-e). Similarly, in HEK293T cells exogenously co-transfected with Myc-tagged CDC7 and HA-tagged STAT3, CDC7 was readily detected in association with STAT3 (Figure 4f). Notably, loss of DBF4 diminished the interaction between CDC7 and STAT3 (Figure 4g). We then determined which conserved domain of STAT3 was responsible for the binding with CDC7. Serial deletion analysis of STAT3 showed that the coiled-coil domain (CCD) of STAT3 was essential for the interaction with CDC7 (Figure 4h). On the other hand, ectopic expression of Flag-tagged DBF4 enhanced the interaction between STAT3 and CDC7, while a catalytic inactive mutant CDC7^D196N^ lost its ability to bind to STAT3 (Figure 4i), suggesting that catalytic activity of CDC7 is critical for its interplay with STAT3. Furthermore, in contrast to wild-type CDC7, ectopic expression of CDC7^D196N^ mutant showed little effect to increase STAT3 phosphorylation or promote HCC cell proliferation (Figure 4j-l).

### XPO1 directs DDK nuclear export and facilitates STAT3 signaling activation

**Figure 5:**
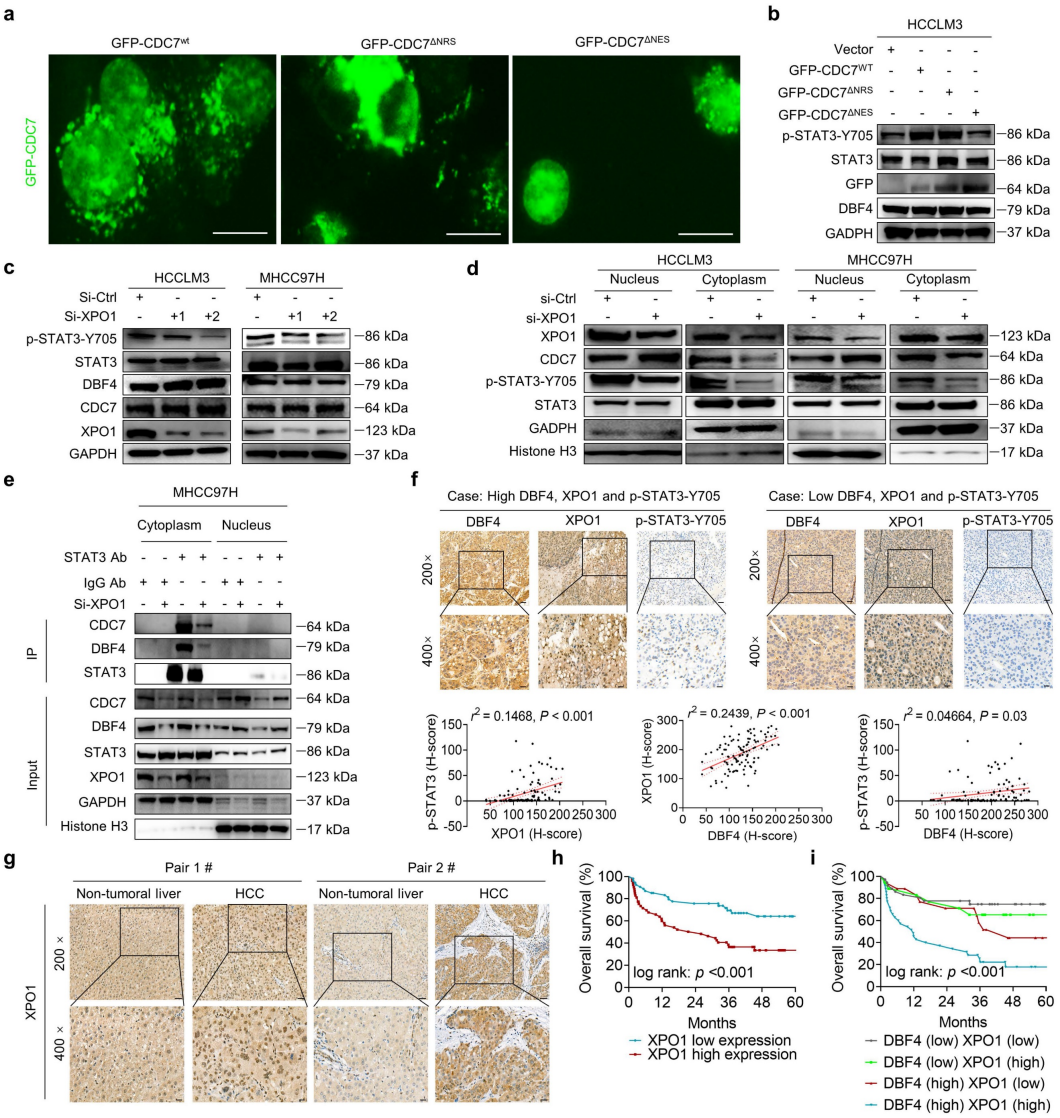
** XPO1 promotes DDK nuclear export and STAT3 pathway activation in HCC cells.** (a). The localization of CDC7 (labeled by GFP) in HCCLM3 cells transfected with different CDC7 expression vectors. Scale bars: 20 μm. (b). Western blotting was conducted to detect the abundance of p-STAT3-Y705 in HCCLM3 cells transfected with different CDC7 expression vectors. (c). Western blot analysis of p-STAT3-Y705, CDC7, and DBF4 expression in HCCLM3 and MHCC97H cells transfected with siRNAs targeting XPO1. (d). Cytoplasmic and nuclear proteins from XPO1-sliencing HCC cells were separated and used for immunoblotting analysis with the indicated antibodies. (e). MHCC97H cells were transfected with siRNAs targeting XPO1. Cytoplasmic and nuclear proteins were then separated and subjected to immunoprecipitation as indicated. Histone H3 and GADPH were used as internal references for the nuclear and cytoplasmic fractions, respectively. (f). Representative images and statistical analysis of IHC staining of DBF4, p-STAT3-Y705, and XPO1 expression in clinical HCC tissue samples. Scale bars: 50 μm for 200× magnification and 20 μm for 400× magnification. (g). IHC staining of XPO1 in HCC and non-tumoral liver tissues. Scale bars: 50 μm for 200× magnification and 20 μm for 400× magnification. (h). Kaplan-Meier plots of HCC patient survival stratified by XPO1 expression. (i). The association of the coexpression of DBF4 and XPO1 with patient survival was assessed.

To explore whether DDK activated STAT3 pathway in a subcellular compartment-dependent manner, we transfected HCCLM3 cells with three types of GFP-tagged vectors expressing wild-type CDC7 (CDC7^wt^), CDC7 without NES (del 458-467+545-554 aa, CDC7^∆NES^), or CDC7 without nuclear retention sequence (NRS; deletion of 300-332 aa, CDC7^∆NRS^), respectively.^29^ As a result, the deletion of NES but not of NRS on CDC7 abolished its nuclear export and moreover, attenuated the ability of CDC7 to boost STAT3 phosphorylation (Figure 5a-b).

It was previously shown that XPO1 could bind to DDK and help transport DDK to the cytoplasm.^29^ We speculated whether XPO1 was involved in the DDK-mediated STAT3 activation. Indeed, we found that siRNAs-mediated XPO1 silencing suppressed STAT3 activation, although it had no overt effect on total DDK level (Figure 5c). Similar results were also obtained after treatment with selinexor, a highly selective and covalent XPO1 inhibitor approved by FDA to treat multiple myeloma (Figure S6c). By contrast, ectopic XPO1 expression promoted STAT3 activation, while this effect was abolished upon the depletion of DBF4 (Figure S6d). In addition, the expression of XPO1 positively correlated with that of STAT3 downstream genes according to the TCGA dataset (Figure S6e). Coimmunoprecipitation assay revealed that XPO1 directly interacted with DDK in HCC cells (Figure S6f). Silencing of XPO1 impaired the nuclear export of DDK, leading to cytoplasm loss and nuclear accumulation of DDK (Figure 5d), whereas the reverse was true for ectopic expression of XPO1 (Figure S6g). Furthermore, silencing of XPO1 remarkedly attenuated the endogenous interaction between DDK and STAT3 in the cytosolic fraction of HCC cells (Figure 5e). Therefore, these findings demonstrate that XPO1 mediates the nuclear export of DDK and facilitates STAT3 activation.

To further explore the potential clinical applications of these data, we measured XPO1 level in clinical HCC tissues by an IHC analysis. Strikingly, the abundance of XPO1 was found to be positively correlated with both DBF4 and activated STAT3, in addition to the observation that the expression of DBF4 is parallel with activated STAT3 (Figure 5f). We found that XPO1 expression was upregulated in HCC tissues, which was consistent with previous reports (Figure 5g).^7^ Besides, HCC patients with elevated XPO1 expression showed inferior survival, and moreover, the combination of both DBF4 and XPO1 high expression yielded the worst prognosis (Figure 5h-i).

### P65 induces DBF4 expression by directly binding to its promoter

**Figure 6:**
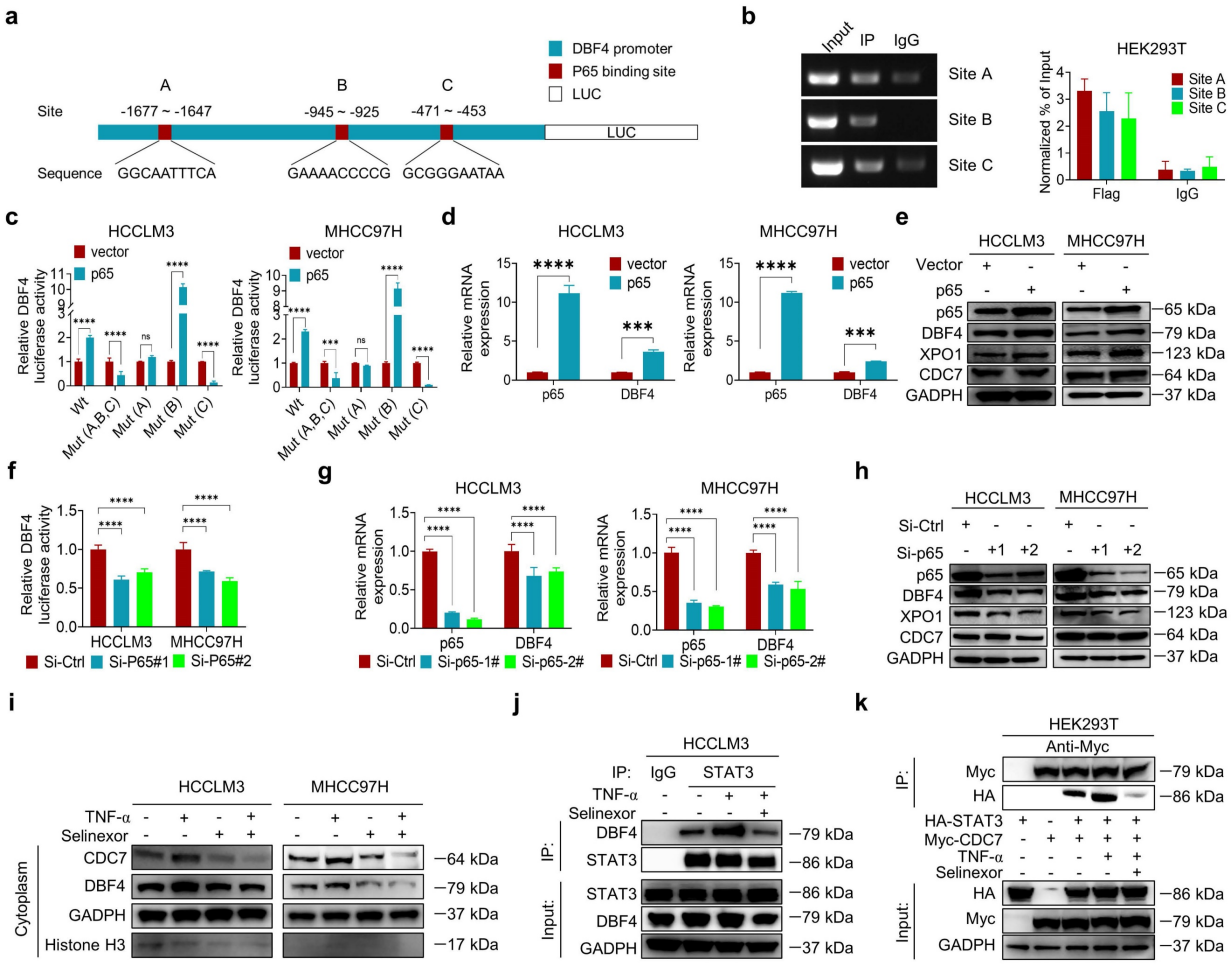
** P65 transcriptionally upregulates DBF4 expression and enhances DDK-STAT3 interaction.** (a). Construction of DBF4 wild-type and deletion mutant promoter luciferase reporter gene system. (b). The interaction between p65 and DBF4 promoter was shown by ChIP assay in HEK293T cells. (c). Dual luciferase assays were used for detecting DBF4 promoter activity after overexpression of p65. (d). RT-qPCR analysis of p65-overexpressing HCC cells as indicated. (e). Expression of p65, DBF4, CDC7 and XPO1 in p65-overexpressing HCC cells was detected by western blotting. (f-h). HCC cells were treated with siRNAs targeting p65. DBF4 promoter activity was measured (f). RNA extracts and cell lysates were subjected to RT-qPCR assays (g) and western blotting (h) as indicated, respectively. (i). HCCLM3 and MHCC97H cells were treated with or without TNF-α (2 ng/mL, 48 hours) and selinexor (1 mM, 72 hours). The cytoplasmic fractions were prepared and subjected to western blotting. (j). HCCLM3 cells were treated with or without TNF-α and selinexor followed by STAT3 co-immunoprecipitation assay. (k). HEK293T cells were transfected with Myc-tagged CDC7 and/or HA-tagged STAT3 and treated with TNF-α and selinexor. Whole cell lysates were immunoprecipitated with anti-Myc antibody, and the precipitates were then analyzed with western blotting with anti-Myc antibody and anti-HA antibody. TNFα, tumor necrosis factor α. ** *P* < 0.01, ***P < 0.001, ****P < 0.0001; ns, not significant.

Considering that DBF4 is elevated in HCC tissues and promotes STAT3 activation, it is worth uncovering the upstream factor that transcriptionally upregulates DBF4 expression. Bioinformatic analysis identified three putative p65 binding sites on DBF4 promoter (-1677 ~ -1647 bp, -945 ~ -925 bp, and -471 ~ -453 bp regions; Figure 6a). ChIP assays revealed the direct binding of p65 to the DBF4 promoter (Figure 6b). Consistently, ectopic p65 expression significantly increased DBF4 promoter activity, mRNA level and protein expression, while the reverses were true for siRNA-mediated silencing of p65 (Figure 6c-h). We confirmed that -1677 ~ -1647 bp and -471 ~ -453 bp regions were essential for p65-induced DBF4 promoter activation in HCC cells (Figure 6c). In further support of these findings, p65 activation by TNF-α stimulation increased DBF4 mRNA and protein expression, while treatment with caffeic acid phenethyl ester (CAPE), a specific p65 inhibitor, was found to reduce DBF4 levels (Figure S7a-d). These data suggest that p65 binds to DBF4 promoter and promotes its transcription.

By serendipity, we observed that ectopic p65 expression simultaneously increased XPO1 mRNA and protein levels in HCC cells (Figure 6e and Figure S8a). Besides, p65-silenced HCC cells showed decreased XPO1 expression (Figure 6h and Figure S8b). However, manipulation of p65 had no significant impact on CDC7 level (Figure 6e, h). We therefore speculated whether p65 modulated XPO1 transcription. We identified three presumptive p65 binding sites in the XPO1 promoter (-1780 ~ -1770 bp, -837 ~ -827 bp, and -272 ~ -262 bp regions; Figure S8c). ChIP assays further verified the direct binding of p65 to the XPO1 promoter (Figure S8d). Consistently, ectopic p65 expression increased whereas silencing p65 decreased the promoter activity of XPO1 (Figure S8e-f). We further revealed that depletion of XOP1 promoter -1780 ~ -1770 bp region largely abolished p65-mediated XPO1 Luciferase expression (Figure S8e). These results demonstrated that p65 transcriptionally upregulates XPO1 by directly binding to its promoter.

As p65 regulated XPO1 expression while XPO1 mediated the nuclear-cytoplasmic shuttle of DDK, we speculated whether p65 would consequently affect the nuclear export of DDK. As expected, TNF-α promoted while CAPE inhibited the nuclear export of DDK in HCC cells (Figure S8g). TNF-α stimulation led to an increase in the abundance of p-STAT3-Y705, whereas DBF4 depletion was shown to attenuate TNF-α-induced upregulation of p-STAT3-Y705 (Figure S8h). Besides, TNF-α promoted the nuclear export of DDK and STAT3 downstream gene expression, whereas the addition of selinexor attenuated these effects (Figure 6i and Figure S8i). Furthermore, endogenous and exogeneous interaction between DDK and STAT3 was enhanced upon exposure to TNF-α, while these effects were again attenuated by selinexor (Figure 6j-k). In addition, we noted that the expression level of p65 presented a strongly positive correlation with both that of DBF4 and XPO1 in both TCGA dataset and clinical samples (Figure S8j-k). Taken together, these results establish a critical role of p65 in enhancing DDK-mediated STAT3 activation through upregulating the transcriptions of DBF4 and XPO1.

### DDK inhibitor XL413 suppresses HCC growth and sensitizes anti-PD-1 immunotherapy

Given the above data supporting the significant role of DDK in facilitating HCC progression, we evaluated whether XL413, a highly selective DDK inhibitor, could be repurposed for the treatment of HCC. We found that XL413 treatment dramatically decreased the proliferating activities and colony-forming capabilities while increased the apoptosis in HCCLM3 and MHCC97H cells (Figure S9a-c). Interestingly, we always observed that XL413 treatment resulted in altered cell morphology under microscope (Figure S9d). In addition, XL413 decreased the phosphorylation of STAT3 and its downstream c-Myc expression in HCC cells, as well as abolished XPO1-mediated STAT3 phosphorylation (Figure S9e-f), providing further evidences on the role of DDK in regulating STAT3 pathway. Notably, XL413 did not significantly impair viability or colony-forming capabilities in non-transformed hepatocytes (Figure S9g-h).

**Figure 7:**
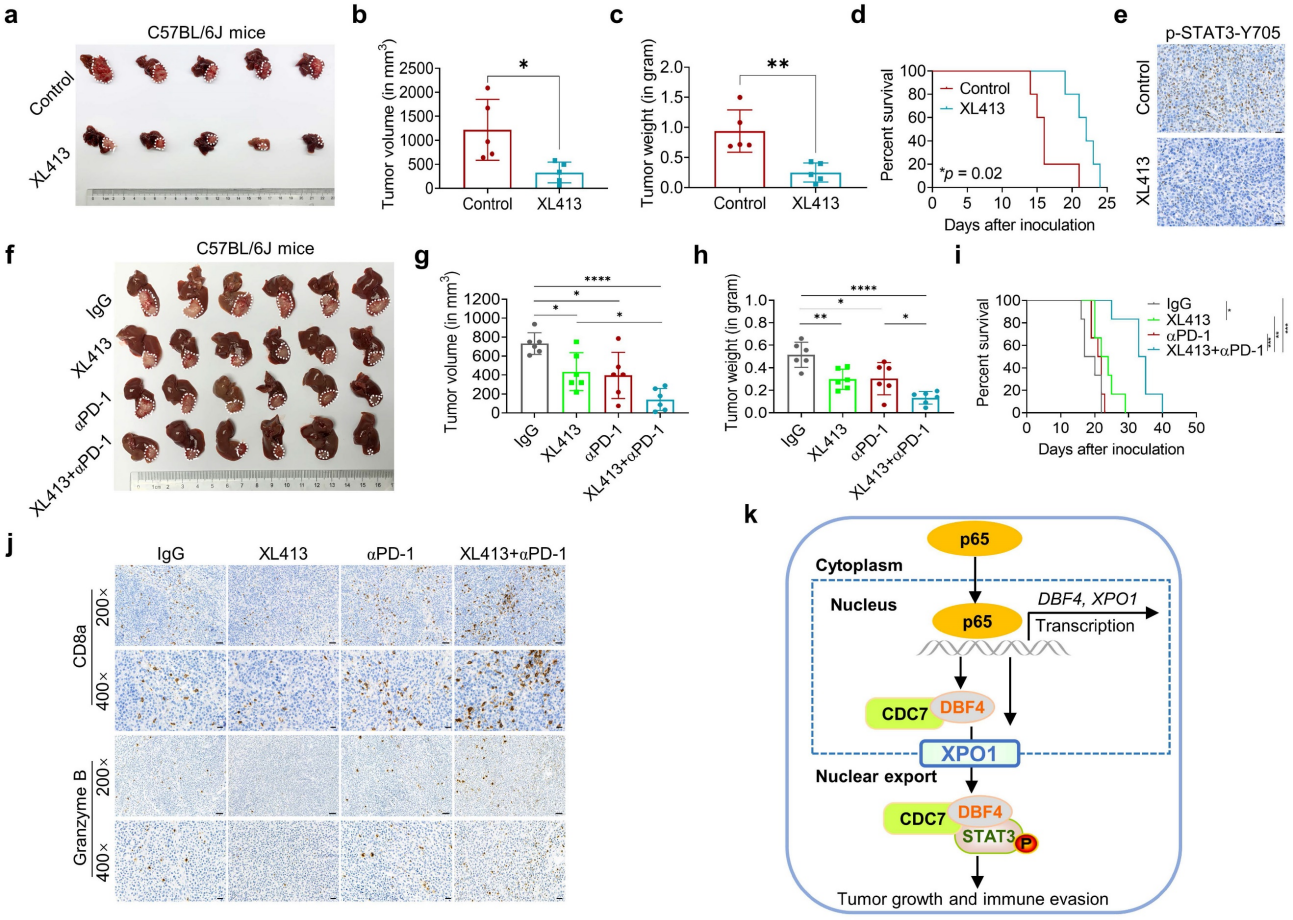
** DDK inhibitor XL413 enhances the therapeutic effects of anti-PD-1 immunotherapy.** (a-e). Hepa1‐6 cells were inoculated into the liver of C57BL/6J mice to establish HCC orthoptic xenograft model (n = 5 for each group). The models were treated with XL413 (50 mg/kg, oral gavage, 6 days per week) or PBS for two weeks. Tumor appearance (a), tumor volume (b) and weight (c), and survival of mice (d). Expression of p-STAT3-Y705 in tumor tissue was detected by IHC staining (e). Scale bars: 20 μm (400× magnification). (f-i). The combination of XL413 (50mg/kg, oral gavage, 6 days per week) and anti-PD-1 antibody treatment (200μg, intraperitoneally administrated, every three days) in orthotopic HCC mouse models. (f). Representative images of Hepa1-6 tumors from each group (per group, n=6). Qualification of tumor volume (g) and weight (h). Survival of mouse xenograft models (i). IHC staining were applied in separated tumors from orthotopic HCC mouse model (j). Scale bars: 50 μm for 200× magnification and 20 μm for 400× magnification. (k). Schematic diagram for the regulatory relationship among p65, DBF4, CDC7, XPO1 and STAT3 in HCC cells. * *P* <0.05, ** *P* < 0.01, ***P < 0.001, ****P < 0.0001; ns, not significant.

We next investigated the therapeutic efficacy of XL413 *in vivo*. In conjunction with the *in vitro* results, treatment with XL413 limited tumor growth and prolonged mouse survival, as well as reduced p-STAT3-Y705 expression in tumor tissues (Figure 7a-e). Furthermore, we did not find any unfavorable toxicity in the mice when treated with XL413, as exemplified by no obvious change in body weight, alanine aminotransferase, aspartate aminotransferase, bilirubin and creatine (Figure S9i-m). Collectively, these findings showed the antitumoral activity and high selectivity towards cancer cells of DDK inhibition.

At present, single therapy only showed very limited efficacy for HCC. Clinical trials have achieved encouraging results with combination therapy in patients with advanced HCC, indicating that combined therapy would be a promising strategy. Because inhibiting STAT3 pathway was recently found to improve the efficacy of anti-PD-1/PD-L1 treatment,^30^, ^31^ we evaluated the combinatorial effect of DDK inhibitor and anti-PD-1 antibody in HCC-bearing murine models. Notably, the application of XL413 with anti-PD-1 antibody demonstrated a significant improvement in tumor burden and mice survival (Figure 7f-i and Figure S10a-c). To further confirm the combination effect of DDK inhibition and PD-1 blockade, we analyzed xenograft tissues by an IHC assay and found markedly increased abundance of tumor-infiltrated CD8^+^ T cells and granzyme B expression while decreased PD-1 expression in the combination therapy group (Figure 7j and Figure S10d). In addition, histopathological analysis showed that the combination regimen did not cause remarkable cytotoxicity in the heart, lung, liver, kidney, or spleen (Figure S10e). These results demonstrated that the combination of DDK inhibition and PD-1/PD-L1 blockade elicited a more effective antitumor efficacy.

## Discussion

In the current study, we demonstrate that DBF4 is elevated in HCC and constitutes an independently negative prognostic factor. P65 increases the expression of DBF4 by directly binding to its promoter region, leading to DDK activating STAT3 pathway in HCC via XPO1-mediated nuclear transportation. Suppressing DBF4 expression or pharmacological inhibition of DDK function limits HCC growth by inactivating STAT3 pathway. Treating HCC-bearing mice with DDK inhibitor selectively kills tumoral cells and moreover, improves the therapeutic efficacy of anti-PD-1 treatment. Our study provides evidences on the contributing role of DDK-STAT3 axis to HCC progression and suggests that DDK may be an excellent therapeutic target for the treatment of HCC.

As a point of convergence for numerous oncogenic signaling pathways, STAT3 is widely involved in HCC progression, as which is constitutively activated in nearly 60% of HCC and associated with poor prognosis.^32^ Fairly well-characterized, persistently activated STAT3 promotes tumor cell proliferation, survival, migration, invasion, and angiogenesis by upregulating downstream target gene expression. Activated STAT3 has also shown to help evade immune surveillance and negatively influence the efficacy of immunotherapy.^30^ A plethora of factors has been suggested to regulate STAT3 activity in cancer cells. Recently, we showed that ASGR1 as a tumor suppressor could inhibit STAT3 activation by interacting with NLK in HCC cells.^25^ The current study reported the identification of DBF4 as another important factor to modulate STAT3 activity. We observed that DBF4 positively correlated with activated STAT3 in HCC tissues. In addition, loss of DBF4 inhibits STAT3 phosphorylation in HCC cells in the presence or absence of IL-6 stimulation. Mechanistic findings further revealed that DBF4 activated STAT3 signaling by recruiting CDC7 to bind to the CCD of STAT3. The CCD at the amino terminus has been implied to be essential for protein-protein interaction, receptor recruitment, and tyrosine phosphorylation of STAT3.^33^ Intriguingly, a recent study by Zhou et al reported that a lncRNA-encoded polypeptide ASRPS could bind to the CCD of STAT3 and downregulate STAT3 phosphorylation, thereby inhibiting angiogenesis in breast cancer.^34^ Thus, these results indicate that effects of the CCD on STAT3 activity might depend on the specific tissue context and different interacting molecules.

As described earlier, XPO1 belongs to the importin-β superfamily of karyopherins and is responsible for the nucleocytoplasmic shuttling of a wide-range of cargoes. Intriguingly, XPO1 directly binds to CDC7, and this binding leads to its translocation to the cytoplasm.^29^, ^35^, ^36^ However, the specific cellular context and biological consequence of this nucleocytoplasmic trafficking remain largely unclear. In our attempts to identify p65 as an upstream inducer which transcriptionally upregulates DBF4, we found that p65 enhanced the transcription and expression of XPO1 by binding to XPO1 promoter, and through which p65 facilitated the transport of DDK from nucleus to cytoplasm. Therefore, these findings suggest that XPO1-mediated nuclear export of DDK is likely controlled by p65. In addition, we demonstrated that XPO1-mediated nuclear export of DDK resulted in enhanced STAT3 activation, indicating STAT3 as a downstream effector of DDK-XPO1 interplay. Together with prior observations, our findings may support a model in which p65-induced XPO1 transports DDK into the cytoplasm, where DDK binds to and activates STAT3 (Figure 7k). As appreciated from this model, XPO1 has a bridging and feedforward role in DDK-mediated STAT3 signaling upon p65 transcriptional activation. In this regard, either deletion in NES of CDC7 or silencing XPO1 expression abrogated the nuclear exportation of CDC7 and consequently inhibited STAT3 pathway. By contrast, p65 upregulated XPO1 expression, promoted the cytoplasm localization of DDK, and enhanced the interaction between DDK and STAT3. As confirmation, DBF4 expression showed positive correlation with XPO1 upregulation in clinical HCC tissues. Moreover, increased level of DBF4 or XPO1 negatively affects HCC patient survival, and the coexpression of DBF4 and XPO1 renders the worst prognosis, providing further clinical implications.

With increasing evidences highlighting the potential of DDK as a target for cancer therapy, a handful of agents including PHA-767491, XL413, TAK-931 and LY3178399 have been introduced to inhibit DDK function.^9^, ^10^ PHA-767491, as the first nanomolar, ATP-competitive, DDK small molecule inhibitor, indeed displayed anti-tumour activity in several pre-clinical models. However, subsequent assays observed the off-target effects of PHA-767491 on CDK9 and RNA polymerase II phosphorylation. XL413 is a potent and selective DDK inhibitor, and it displayed good pharmacokinetic profile and anti-tumor activity in previous studies.^37^ XL413 also fits well into the selective cytotoxicity of DDK inhibitors against tumor cells. In fact, a recent study by Wang et al has documented that XL413 treatment alone could selectively impair the proliferation of HCC cells *in vitro* and *in vivo*.^38^ Moreover, XL413 was found to collaborate with mTOR inhibitor to drastically inhibit HCC growth by blocking feedback re-activation of mTOR signaling, delivering so called “one-two-punch” strategy. In another study, XL413 was implied to potentiate ATR-CHK1 signaling inhibition in HCC.^38^ However, the therapeutic efficacy of combining DDK inhibitor and immune checkpoint blockade has not yet been investigated so far. The present study ascertained the property of XL413 to highly selectively kill HCC cells. Furthermore, our study revealed that XL413 enhanced the efficacy of anti-PD-1 immunotherapy, offering new options for the treatment of HCC. Further studies will be needed to elucidate the potential role of DDK in cancer immune resistance. In addition, it is also worth exploring the efficacy of other DDK inhibitors to broad potential of targeting DDK for the treatment of HCC.

## Conclusions

In summary, our study reveals that DBF4, characterized as a pro-oncogenic factor, promotes STAT3 signaling activation via a XPO1-dependent manner mediated by CDC7 in HCC cells. Furthermore, combining DDK inhibitor treatment with anti-PD-1 immunotherapy may be a promising option to improve the therapeutic efficacy for HCC.

## Supplementary Material

Supplementary figures and tables.

## Figures and Tables

**Table 1:** Correlation between DBF4 expression and clinicopathologic variables in 107 HCC patients.

Clinicopathological characteristics		All subjects (n = 107)	DBF4 low expression (n = 54)	DBF4 high expression (n = 53)	*P*
Age (years)		54.6 ± 12.7	53.3 ± 13.5	56.0 ± 11.8	0.27
Gender	Male	100 (93.5%)	50 (92.6%)	50 (94.7%)	0.72
	Female	7 (6.5%)	4 (7.4%)	3 (5.7%)	
HBV infection	With	88 (82.2%)	43 (79.6%)	45 (84.9%)	0.48
	Without	19 (17.8%)	11 (20.4%)	8 (15.1%)	
Underlying cirrhosis	Present	87 (81.3%)	46 (85.2%)	41 (77.4%)	0.30
	Absent	20 (18.7%)	8 (14.8%)	12 (22.6%)	
Serum AFP level	≤400 ng/mL	67 (62.6%)	31 (57.4%)	36 (67.9%)	0.26
	>400 ng/mL	40 (37.4%)	23 (42.6%)	17 (32.1%)	
No. of nodules	Solitary	87 (81.3%)	45 (83.3%)	42 (79.2%)	0.59
	Multiple	20 (18.7%)	9 (16.7%)	11 (20.8%)	
Tumor size	≤5cm	32 (29.9%)	22 (40.7%)	10 (18.9%)	0.13
	>5cm	75 (70.1%)	32 (59.3%)	43 (81.1%)	
Tumor differentiation	Grading I-II	76 (71.0%)	38 (70.4%)	38 (71.7%)	0.88
	Grading II-IV	31 (29.0%)	16 (29.6%)	15 (29.0%)	
Microvascular invasion	Present	42 (39.3%)	20 (37%)	22 (41.5%)	0.64
	Absent	65 (60.7%)	34 (63.0%)	31 (58.5%)	

## Abbreviations

CAPE: caffeic acid phenethyl ester
CDC7: cell division cycle 7
ChIP: chromatin immunoprecipitation
DBF4: dumbbell former 4
DDK: Dbf4-dependent kinase
HCC: hepatocellular carcinoma
IHC: immunohistochemistry
IF: immunofluorescence
GAPDH: Glyceraldehyde-3-phosphate dehydrogenase
GFP: green fluorescent protein
NES: nuclear export signal
NRS: nuclear retention sequence
PBS: phosphate buffered saline
PD-1: programmed cell death 1
PD-L1: programmed cell death-ligand 1
STAT3: signal transducer and activator of transcription 3
TCGA: The Cancer Genome Atlas
TNFα: tumor necrosis factor α
XPO1: exportin 1
