# Supplementary material for: DBF4 Dependent Kinase Inhibition Suppresses Hepatocellular Carcinoma Progression and Potentiates Anti-Programmed Cell Death-1 Therapy
Source: Int J Biol Sci. 2023 Jul 3;19(11):3412–27. doi: 10.7150/ijbs.80351 (PMC10367558; doi:10.7150/ijbs.80351)
Supplement: Supplementary file 1 — Supplementary figures and tables. [file ijbsv19p3412s1.pdf]

**Supplementary Figure Legends**

**Figure S1. Negative controls for IF IHC staining.** Stained section without the primary antibody was used as a negative control for DBF4 IHC staining in HCC tissues. Scale bars: 20 μm (400× magnification).

**Figure S2. DBF4 expression is upregulated in HCC tissues and portends inferior clinical outcome.** (a). DBF4 expression in HCC tissues according to tumor T classification in the TCGA dataset. (b). Kaplan-Meier survival curves stratified by DBF4 expression level were generated for HCC patients in the TCGA dataset. (c). Univariate and multivariate Cox proportional hazards analyses were used to determine the prognostic value of DBF4 expression level for HCC patients in the TCGA dataset. Hazard ratio, HR. $^{**}P < 0.01$. $^{****}P < 0.0001$.

**Figure S3. Knockdown of DBF4 induces HCC cell cycle arrest.** Cell cycle analysis of HCCLM3, MHCC97H, Huh7 and SNU449 cells after DBF4 knockdown.

**Figure S4. DBF4 promotes HCC proliferation and tumorigenic ability *in vitro* and *in vivo*.** (a). CCK-8 assays were conducted to evaluate the effect of DBF4 overexpression on the proliferative activities of HCCLM3 and MHCC97H cells. (b). The effect of DBF4 overexpression on the colony forming capabilities of HCC cells. (c). Flow cytometric assay was performed to detect the effect of DBF4 overexpression on the viability of HCC cells. (d). The effect of DBF4 overexpression on HCC cell cycle progression was determined the flow cytometry analysis. (e). IHC evaluation of Ki-67 expression in the HCC subcutaneous xenograft tumorigenesis models in immunodeficient nude mice. Scale bars: 50 μm. (f). The HCC subcutaneous xenograft

tumorigenesis models were established in immunocompetent C57BL/6J mice using Hepa1-6 cells with or without DBF4 knockdown (KD; for each group, n = 5). Macroscopic examination of tumor xenografts (left panel) and western blot analyses (middle panel). Qualifications of tumor volume (upper right panel) and weight (lower right panel). (g). IHC evaluation of Ki-67 expression in subcutaneous (left panel) and orthotopic (right panel) xenografts from DBF4-depleted Hepa1-6 cells. Scale bars: 50 μm for 200× magnification and 20 μm for 400× magnification. * $P$ <0.05, ** $P$ < 0.01, ***$P$ < 0.001, ****$P$ < 0.0001.

**Figure S5. DBF4 promotes STAT3 signaling activation.** (a). The top ten gene sets according to the normalized enrichment score (NES) from the GSEA analysis for the difference in pathway composition between $DBF4^{high}$ and $DBF4^{low}$ HCC samples. (b). DBF4-overexpressing HCCLM3 and MHCC97H cells were lysed and analyzed by western blotting using the indicated antibodies. (c). The abundance of p-STAT3-Y705 was examined by IHC staining in DBF4-depelted Hepa1-6 subcutaneous xenograft tumor tissues. Scale bars: 50 μm for 200× magnification and 20 μm for 400× magnification. (d). Correlations between DBF4 and STAT3 downstream target genes in HCC samples from the TCGA dataset. Correlation coefficients and statistical significances were obtained from the GEPIA dataset using the Spearman's rank-order correlation.

**Figure S6. XPO1-mediated nuclear export is essential for DDK-induced STAT3 activation in HCC.** (a). RT-qPCR assays were used to analyze the several of STAT3 target gene expression levels in HCC cells transfected with siRNAs targeting CDC7.

(b). Western blot was applied in DBF4-overexpressing and CDC7-sliencing HCCLM3 cells using the indicated antibodies. (c). XPO1 inhibitor selinexor was applied in HCCLM3 cells at the indicated concentrations for 72 hours. Expression of DBF4, CDC7, STAT3 and p-STAT3-Y705 was detected by western blotting. (d). Western blotting was used to determine p-STAT3-Y705 expression in XPO1-overexpressing HCCLM3 cells with or without DBF4 depletion. (e). Correlations between XPO1 and STAT3 downstream target genes in HCC samples from the TCGA dataset. (f). Coimmunoprecipitation assay was performed in HCCLM3 and MHCC97H cells to evaluate the interaction between XPO1 and DDK. (g). HCCLM3 cells were transfected with XPO1-overexpressing plasmid and subjected to western blot analysis of cytoplasmic and nuclear proteins.

**Figure S7. p65 upregulates DBF4 transcription in HCC cells.** (a, b). HCCLM3 and MHCC97H cells were treated with TNF-α at indicated concentration for 24 hours. RNA extracts and cell lysates were subjected to RT-qPCR assays (a) and western blotting (b) as indicated, respectively. (c, d). RT-qPCR (c) and western blotting (d) were conducted in HCC cells treated with CAPE at the indicated concentrations for 24 hours. CAPE, caffeic acid phenethyl ester; TNF-α, tumor necrosis factor α. * $P < 0.05$, ***$P < 0.001$, ****$P < 0.0001$; ns, not significant.

**Figure S8. p65 transcriptionally upregulates XPO1 expression and enhances DDK-STAT3 interaction in HCC cells.** (a, b). XPO1 mRNA expression level was detected in HCC cells after overexpression of p65 (a) or inhibition of p65 expression by siRNAs (b). (c). Construction of XPO1 wild-type and deletion mutant promoter

luciferase reporter gene system. (d). ChIP analysis showed the binding of p65 to the XPO1 promoter region. (e, f). XPO1 promoter activity were measured after overexpression of p65 (e) or inhibition of p65 expression by siRNAs (f). (g). HCC cells were treated with CAPE or TNF-α at the indicated concentrations for 24 hours. Cytoplasmic proteins were separated for western blotting analysis using the indicated antibodies. (h). Western blotting was conducted to examine p-STAT3-Y705 expression in DBF4-depleted HCCLM3 upon TNF-α stimulation (1 ng/mL) for 24 hours. (i). RT-qPCR assays were performed to assess several of STAT3 downstream gene expression in HCCLM3 cells treated with or without TNF-α and selinexor. (j). Correlations among p65, DBF4 and XPO1 expression in HCC patients from the TCGA dataset. (k). Correlation analysis among p65, DBF4 and XPO1 expression in 20 cases of clinical HCC tissue samples using western blotting. Protein levels were relative to GADPH. CAPE, caffeic acid phenethyl ester; TNF-α, tumor necrosis factor α. ** $P < 0.01$, ***$P < 0.001$, ****$P < 0.0001$; ns, not significant.

**Figure S9. DDK inhibitor XL413 suppresses HCC progression with minimal toxicity.** (a). Colony formation assay was performed in HCCLM3 and MHCC97H cells cultured with the indicated concentration of XL413. (b). CCK-8 assay was applied to evaluate the proliferative activities of HCC cells treated with XL413 (10 μM). (c). Flow cytometric assay was used to detect the effect of XL413 treatment (10 μM) on the viability of HCC cells. (d). HCC cell morphology under microscope upon exposure to XL413 (10 μM, 48 hours). (e). Expressions of p-STAT3-Y705 and c-Myc in HCC cells were detected by western blotting after treatment with XL413 (10 μM, 48 hours). (f).

Western blotting analysis was performed in XPO1-ovexpressing HCC cells exposed to XL413 (10 μM) for 48 hours. (g) Colony formation assay was conducted in non-transformed cells (HepLi5 and AML-12 cells) cultured with XL413 at the indicated concentrations. (h). HepLi5 and AML-12 cells exposed to 10 μM XL413 for 48 hours were subjected to flow cytometric assay for detecting cell viability. (i-m). Hepa1-6 cells were inoculated into the liver of C57BL/6J mice to establish HCC orthoptic xenograft model (n = 5 for each group). The models were treated with XL413 or PBS for two weeks. Data on body weight was described as mean ± SEM and plotted (i). Serum level of alanine aminotransferase (ALT; j), aspartate aminotransferase (AST; k), total bilirubin (TBIL; l) and creatinine (CREA; m) were measured after exposure to XL413. * $P$ <0.05, ** $P$ < 0.01, ***$P$ < 0.001, ****$P$ < 0.0001; ns, not significant.

**Figure S10. Combination with DDK inhibitor XL413 and anti-PD-1 immunotherapy in HCC.** (a-c). The combination of XL413 (50mg/kg, oral gavage, 6 days per week) and anti-PD-1 antibody treatment (200μg, intraperitoneally administrated, every three days) in subcutaneous HCC models. Representative images of Hepa1-6 subcutaneous (a) tumors from each group (per group, n=6). Qualification of tumor volume (b) and weight (c). (d). IHC staining was performed to detect PD-1 and CD44 expression in tumoral tissue sections. (e). Representative H&E staining of the heart, liver, spleen, lung and kidney of the mouse. Scale bars: 100 μm. * $P$ <0.05, ** $P$ < 0.01, ***$P$ < 0.001, ****$P$ < 0.0001.

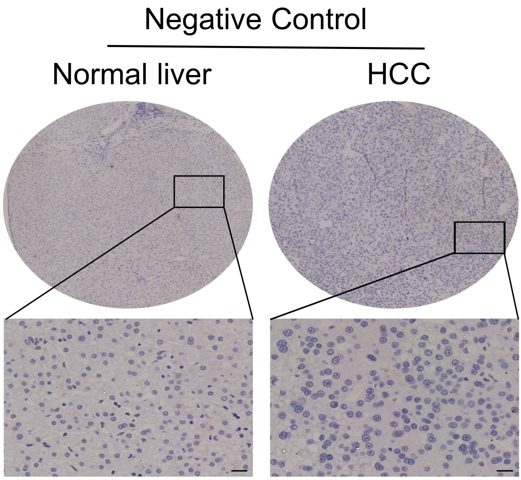

Negative Control
Normal liver
HCC

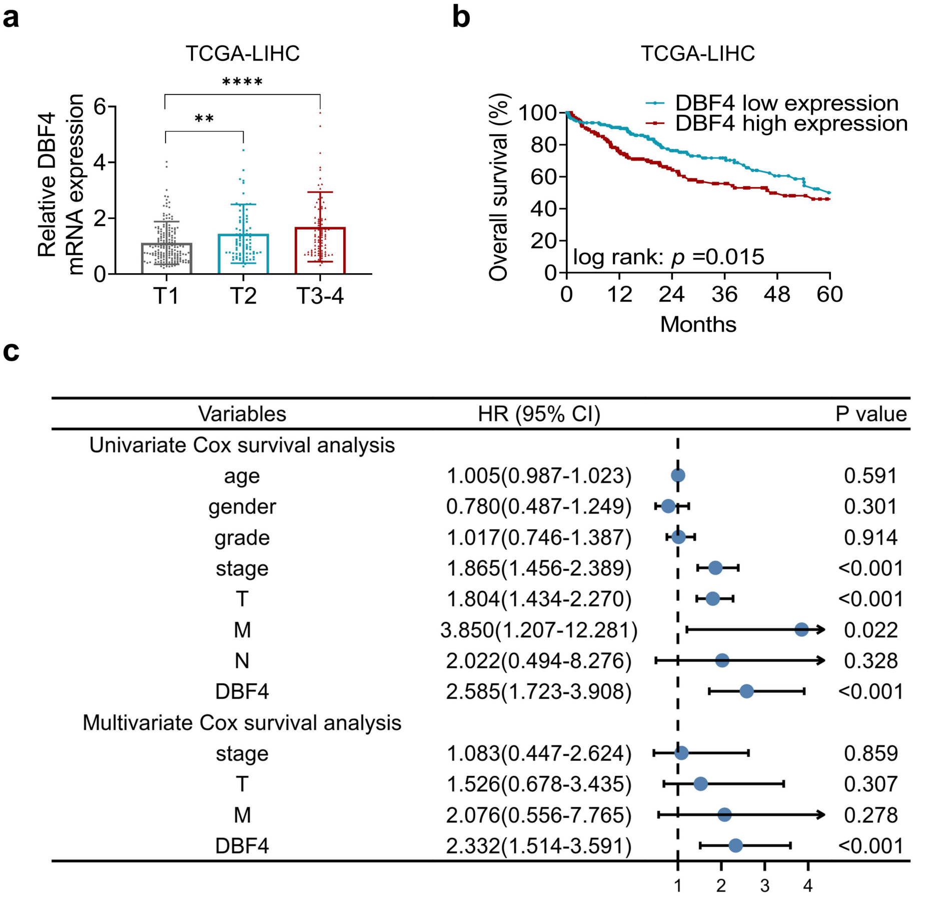
a
TCGA-LIHC
Relative DBF4 mRNA expression
****
**
0
2
4
6
T1
T2
T3-4
b
TCGA-LIHC
DBF4 low expression
DBF4 high expression
Overall survival (%)
0
20
40
60
80
100
log rank: p =0.015
0
12
24
36
48
60
Months
c
Variables
HR (95% CI)
P value
Univariate Cox survival analysis
age 1.005(0.987-1.023) 0.591
gender 0.780(0.487-1.249) 0.301
grade 1.017(0.746-1.387) 0.914
stage 1.865(1.456-2.389) <0.001
T 1.804(1.434-2.270) <0.001
M 3.850(1.207-12.281) 0.022
N 2.022(0.494-8.276) 0.328
DBF4 2.585(1.723-3.908) <0.001
Multivariate Cox survival analysis
stage 1.083(0.447-2.624) 0.859
T 1.526(0.678-3.435) 0.307
M 2.076(0.556-7.765) 0.278
DBF4 2.332(1.514-3.591) <0.001
1
2
3
4

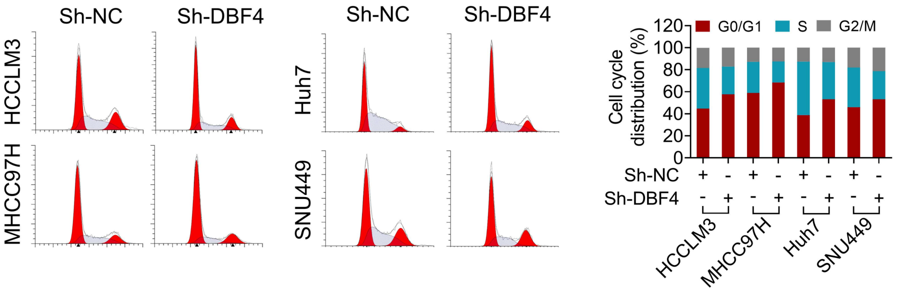

Sh-NC
Sh-DBF4
HCCLM3
MHCC97H
Sh-NC
Sh-DBF4
Huh7
SNU449
G0/G1
S
G2/M
Cell cycle distribution (%)
120
100
80
60
40
20
0
Sh-NC
Sh-DBF4
HCCLM3
MHCC97H
Huh7
SNU449

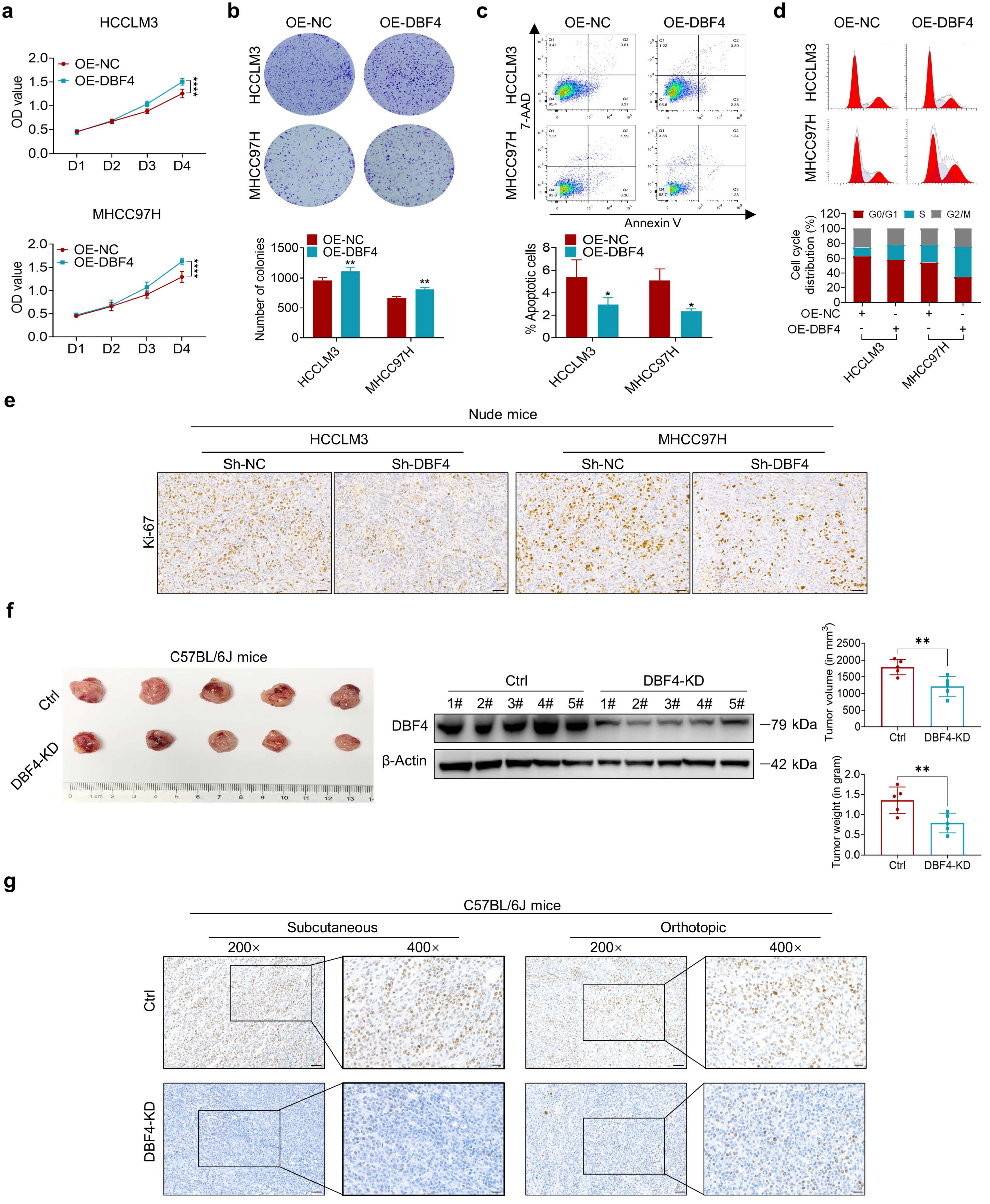
a
HCCLM3
OE-NC
OE-DBF4
OD value
D1
D2
D3
D4
****
MHCC97H
OE-NC
OE-DBF4
OD value
D1
D2
D3
D4
****
b
OE-NC
OE-DBF4
HCCLM3
MHCC97H
OE-NC
OE-DBF4
Number of colonies
**
**
HCCLM3
MHCC97H
c
OE-NC
OE-DBF4
HCCLM3
MHCC97H
7-AAD
Annexin V
OE-NC
OE-DBF4
% Apoptotic cells
*
*
HCCLM3
MHCC97H
d
OE-NC
OE-DBF4
HCCLM3
MHCC97H
G0/G1
S
G2/M
Cell cycle distribution (%)
OE-NC
OE-DBF4
HCCLM3
MHCC97H
e
Nude mice
HCCLM3
MHCC97H
Sh-NC
Sh-DBF4
Sh-NC
Sh-DBF4
Ki-67
f
C57BL/6J mice
Ctrl
DBF4-KD
Ctrl
DBF4-KD
1#
2#
3#
4#
5#
1#
2#
3#
4#
5#
DBF4
β-Actin
—79 kDa
—42 kDa
Tumor volume (in mm³)
**
Ctrl
DBF4-KD
Tumor weight (in gram)
**
Ctrl
DBF4-KD
g
C57BL/6J mice
Subcutaneous
Orthotopic
200×
400×
200×
400×
Ctrl
DBF4-KD

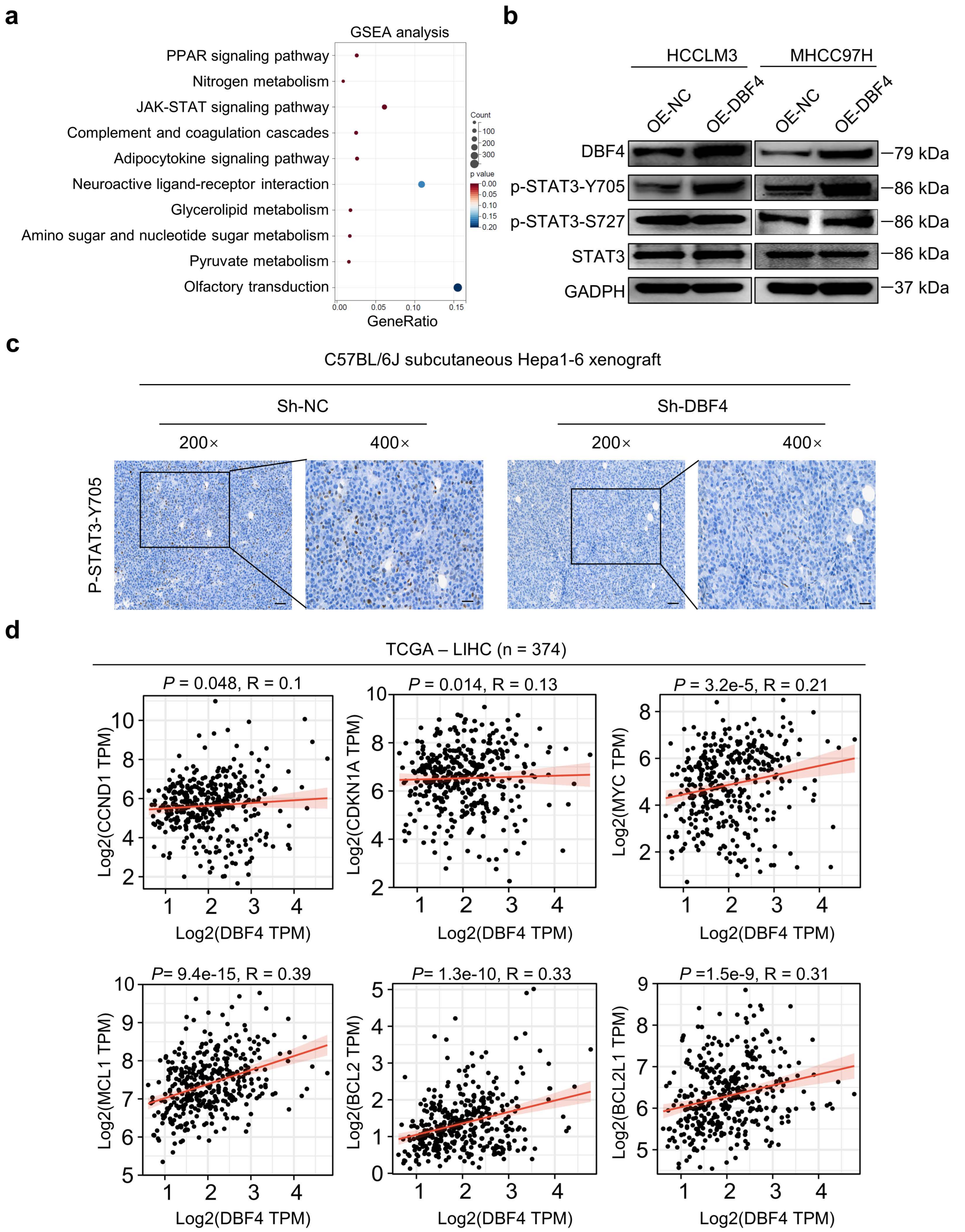

a
GSEA analysis
PPAR signaling pathway
Nitrogen metabolism
JAK-STAT signaling pathway
Complement and coagulation cascades
Adipocytokine signaling pathway
Neuroactive ligand-receptor interaction
Glycerolipid metabolism
Amino sugar and nucleotide sugar metabolism
Pyruvate metabolism
Olfactory transduction
Count
p value
GeneRatio
b
HCCLM3
MHCC97H
OE-NC
OE-DBF4
DBF4
p-STAT3-Y705
p-STAT3-S727
STAT3
GADPH
79 kDa
86 kDa
37 kDa
c
C57BL/6J subcutaneous Hepa1-6 xenograft
Sh-NC
Sh-DBF4
200×
400×
P-STAT3-Y705
d
TCGA – LIHC (n = 374)
P = 0.048, R = 0.1
P = 0.014, R = 0.13
P = 3.2e-5, R = 0.21
P= 9.4e-15, R = 0.39
P= 1.3e-10, R = 0.33
P =1.5e-9, R = 0.31
Log2(CCND1 TPM)
Log2(CDKN1A TPM)
Log2(MYC TPM)
Log2(MCL1 TPM)
Log2(BCL2 TPM)
Log2(BCL2L1 TPM)
Log2(DBF4 TPM)

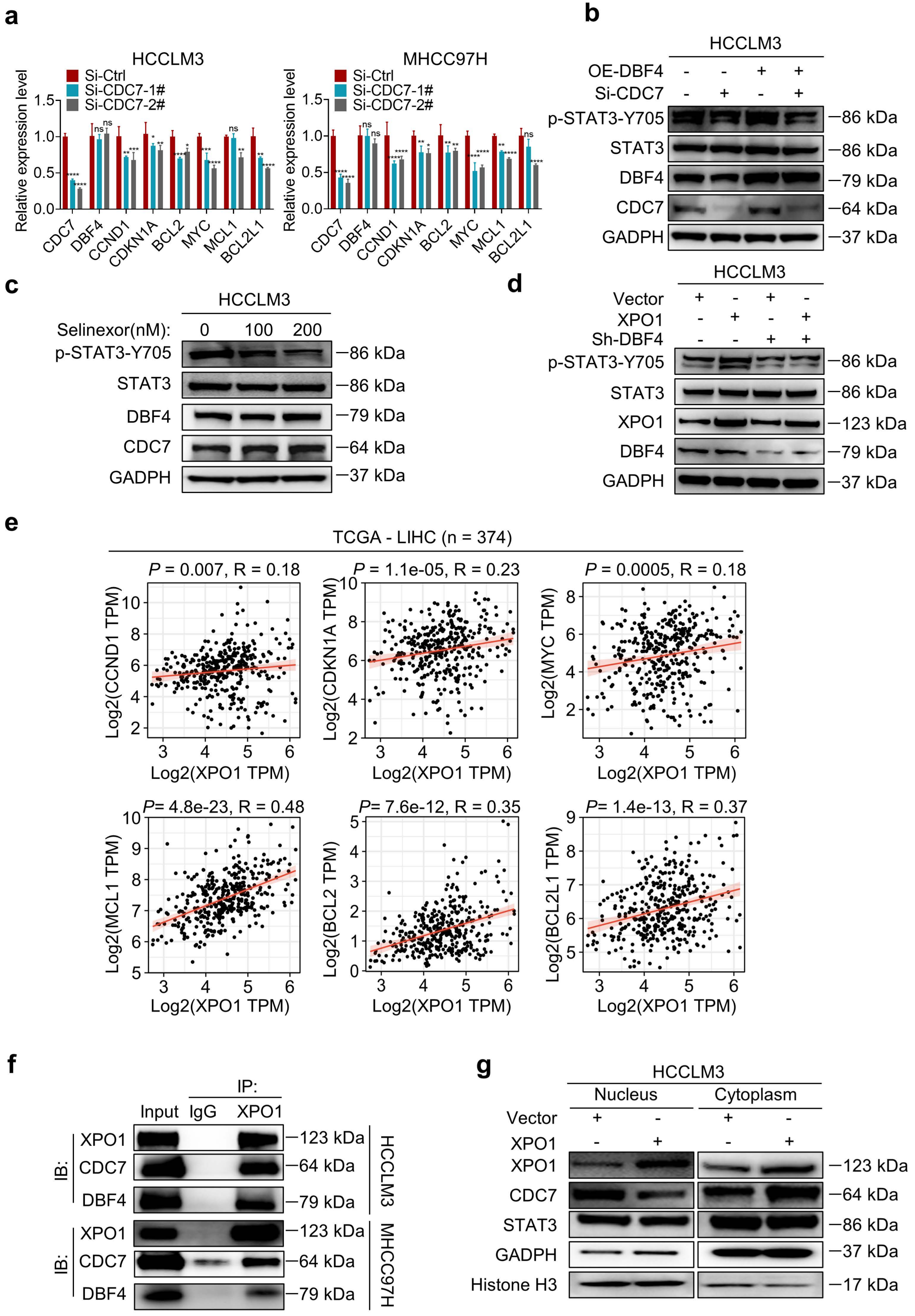
a
HCCLM3
MHCC97H
Si-Ctrl
Si-CDC7-1#
Si-CDC7-2#
Relative expression level
CDC7
DBF4
CCND1
CDKN1A
BCL2
MYC
MCL1
BCL2L1
b
HCCLM3
OE-DBF4
Si-CDC7
p-STAT3-Y705
STAT3
DBF4
CDC7
GADPH
86 kDa
79 kDa
64 kDa
37 kDa
c
HCCLM3
Selinexor(nM): 0 100 200
d
Vector
XPO1
Sh-DBF4
123 kDa
e
TCGA - LIHC (n = 374)
P = 0.007, R = 0.18
P = 1.1e-05, R = 0.23
P = 0.0005, R = 0.18
P= 4.8e-23, R = 0.48
P= 7.6e-12, R = 0.35
P= 1.4e-13, R = 0.37
Log2(CCND1 TPM)
Log2(CDKN1A TPM)
Log2(MYC TPM)
Log2(MCL1 TPM)
Log2(BCL2 TPM)
Log2(BCL2L1 TPM)
Log2(XPO1 TPM)
f
IP:
Input
IgG
XPO1
IB:
HCCLM3
MHCC97H
g
Nucleus
Cytoplasm
Histone H3
17 kDa

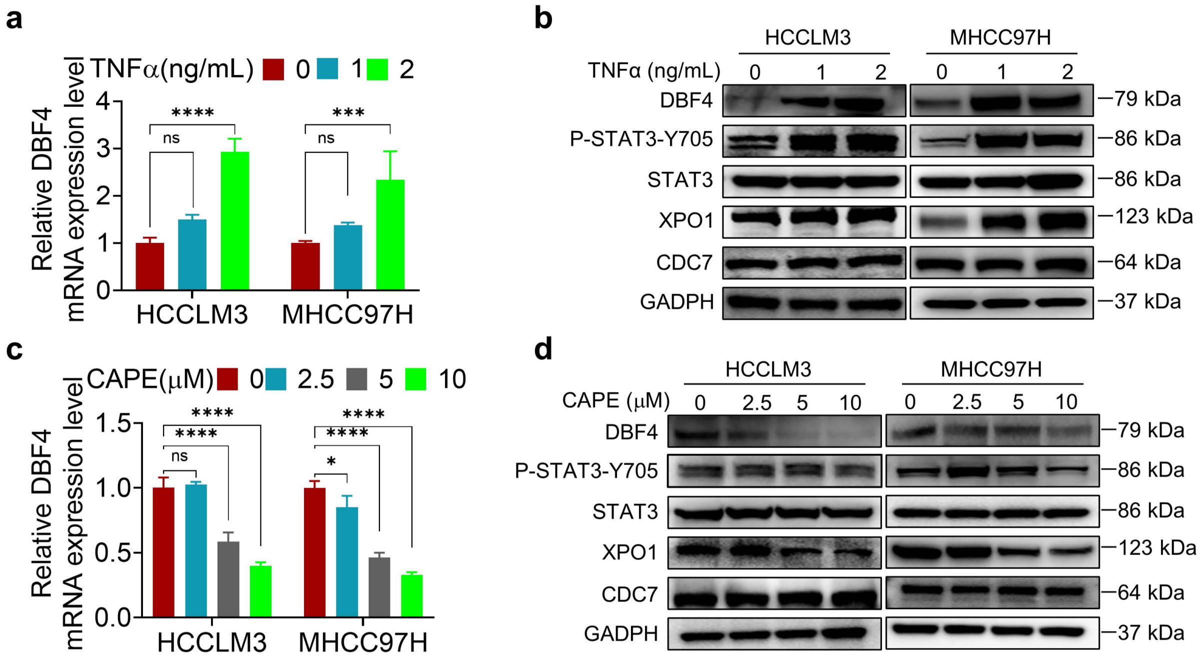
a
TNFα(ng/mL) 0 1 2
Relative DBF4 mRNA expression level
****
***
ns
ns
4
3
2
1
0
HCCLM3
MHCC97H
b
HCCLM3
MHCC97H
TNFα (ng/mL) 0 1 2 0 1 2
DBF4
P-STAT3-Y705
STAT3
XPO1
CDC7
GADPH
79 kDa
86 kDa
86 kDa
123 kDa
64 kDa
37 kDa
c
CAPE(μM) 0 2.5 5 10
Relative DBF4 mRNA expression level
****
****
ns
****
****
*
1.5
1.0
0.5
0.0
HCCLM3
MHCC97H
d
HCCLM3
MHCC97H
CAPE (μM) 0 2.5 5 10 0 2.5 5 10
DBF4
P-STAT3-Y705
STAT3
XPO1
CDC7
GADPH
79 kDa
86 kDa
86 kDa
123 kDa
64 kDa
37 kDa

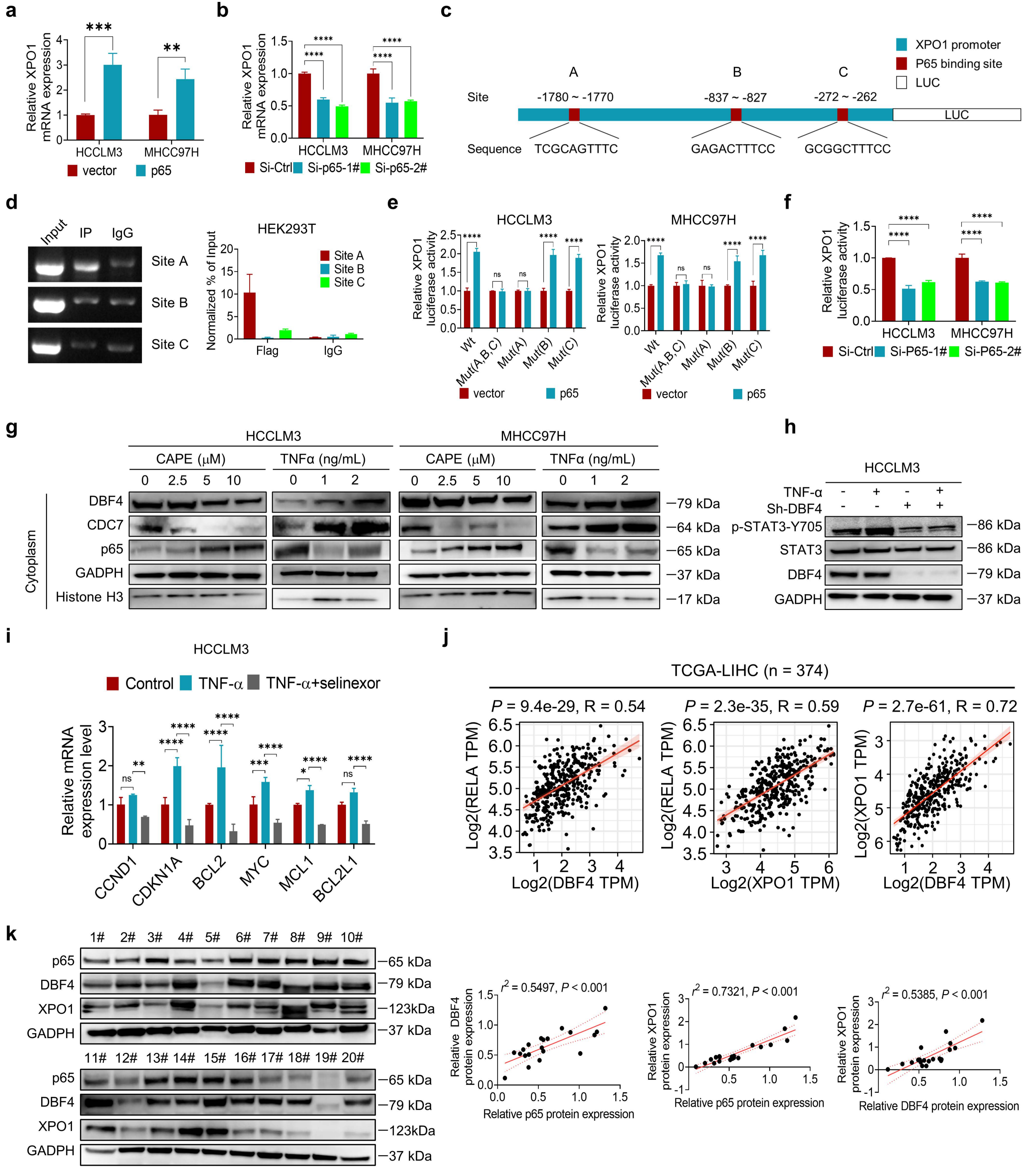

a
Relative XPO1 mRNA expression
HCCLM3
MHCC97H
vector
p65
b
Relative XPO1 mRNA expression
HCCLM3
MHCC97H
Si-Ctrl
Si-p65-1#
Si-p65-2#
c
XPO1 promoter
P65 binding site
LUC
Site
A
B
C
-1780 ~ -1770
-837 ~ -827
-272 ~ -262
Sequence
TCGCAGTTTC
GAGACTTTCC
GCGGCTTTCC
d
Input
IP
IgG
Site A
Site B
Site C
HEK293T
Normalized % of Input
Flag
IgG
e
HCCLM3
MHCC97H
Relative XPO1 luciferase activity
Wt
Mut(A,B,C)
Mut(A)
Mut(B)
Mut(C)
vector
p65
f
Relative XPO1 luciferase activity
HCCLM3
MHCC97H
Si-Ctrl
Si-P65-1#
Si-P65-2#
g
HCCLM3
MHCC97H
CAPE (μM)
TNFα (ng/mL)
0 2.5 5 10
0 1 2
Cytoplasm
DBF4
CDC7
p65
GADPH
Histone H3
—79 kDa
—64 kDa
—65 kDa
—37 kDa
—17 kDa
h
HCCLM3
TNF-α
Sh-DBF4
p-STAT3-Y705
STAT3
DBF4
GADPH
—86 kDa
—86 kDa
—79 kDa
—37 kDa
i
HCCLM3
Control
TNF-α
TNF-α+selinexor
Relative mRNA expression level
CCND1
CDKN1A
BCL2
MYC
MCL1
BCL2L1
j
TCGA-LIHC (n = 374)
P = 9.4e-29, R = 0.54
P = 2.3e-35, R = 0.59
P = 2.7e-61, R = 0.72
Log2(RELA TPM)
Log2(DBF4 TPM)
Log2(XPO1 TPM)
k
1# 2# 3# 4# 5# 6# 7# 8# 9# 10#
11# 12# 13# 14# 15# 16# 17# 18# 19# 20#
p65
DBF4
XPO1
GADPH
—65 kDa
—79 kDa
—123kDa
—37 kDa
r2 = 0.5497, P < 0.001
r2 = 0.7321, P < 0.001
r2 = 0.5385, P < 0.001
Relative DBF4 protein expression
Relative XPO1 protein expression
Relative p65 protein expression
Relative DBF4 protein expression

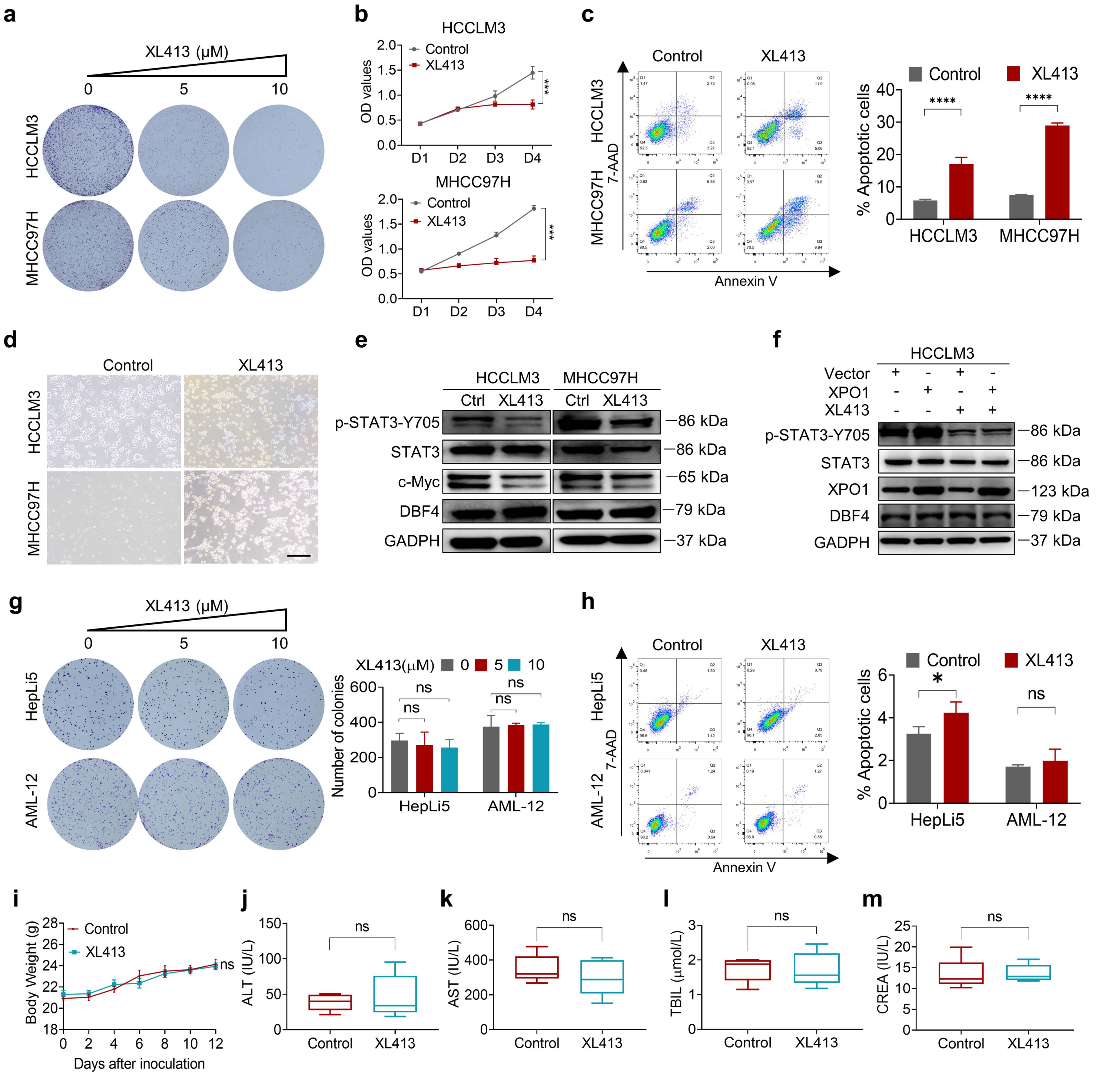

a
XL413 (μM)
0
5
10
HCCLM3
MHCC97H
b
HCCLM3
Control
XL413
OD values
D1
D2
D3
D4
***
MHCC97H
c
Control
XL413
7-AAD
Annexin V
% Apoptotic cells
****
d
e
Ctrl
p-STAT3-Y705
STAT3
c-Myc
DBF4
GADPH
86 kDa
65 kDa
79 kDa
37 kDa
f
Vector
XPO1
123 kDa
g
XL413(μM)
Number of colonies
ns
HepLi5
AML-12
h
*
i
Body Weight (g)
Days after inoculation
j
ALT (IU/L)
k
AST (IU/L)
l
TBIL (μmol/L)
m
CREA (IU/L)

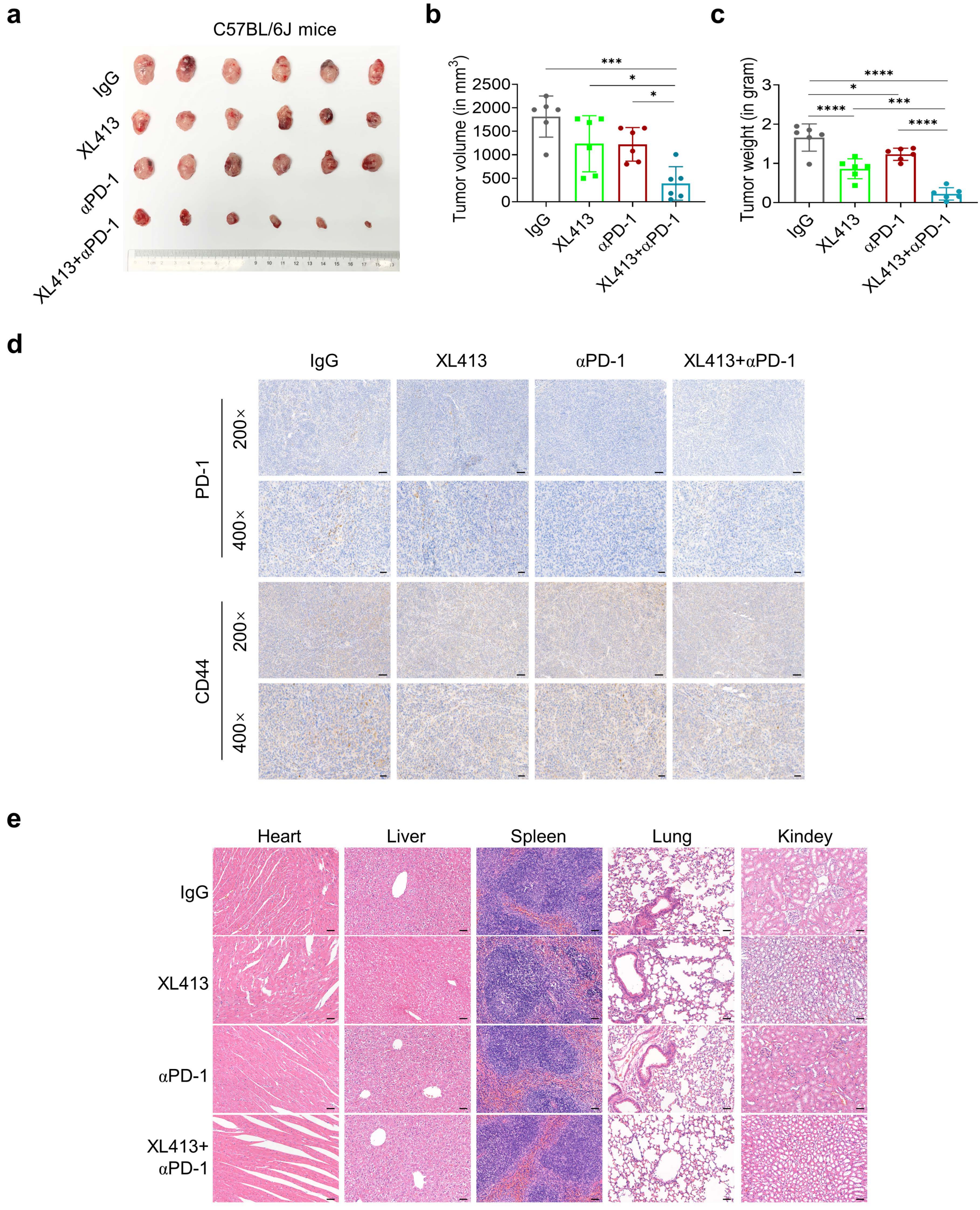

a
C57BL/6J mice
IgG
XL413
αPD-1
XL413+αPD-1
b
Tumor volume (in mm$^3$)
2500
2000
1500
1000
500
0
***
*
*
IgG
XL413
αPD-1
XL413+αPD-1
c
Tumor weight (in gram)
3
2
1
0
****
*
****
***
****
IgG
XL413
αPD-1
XL413+αPD-1
d
IgG
XL413
αPD-1
XL413+αPD-1
PD-1
200×
400×
CD44
200×
400×
e
Heart
Liver
Spleen
Lung
Kindey
IgG
XL413
αPD-1
XL413+
αPD-1

**Supplementary Tables**

**Table S1: shRNA and siRNA sequences used in this study**

| **Target genes** | **Sense (5'-3)'** | **Antisense (5'-3')** |
|---|---|---|
| The sequences of shRNA target | | |
| human-DBF4 | GAATCCAAACAGATGGCGATA | TATCGCCATCTGTTTGGATTC |
| mouse-DBF4 | GCTGAACCGAGTGCTGAATTG | CAATTCAGCACTCGGTTCAGC |
| The sequences of siRNA target | | |
| human-CDC7-1# | CAGCUUAGUAAUGUGUUUATT | UAAACACAUUACUAAGCUGTT |
| human-CDC7-2# | CCAGAGGUCUUGACAAAGUTT | ACUUUGUCAAGACCUCUGGTT |
| human-XPO1-1# | CCAGCAAAGAAUGGCUCAATT | UUGAGCCAUUCUUUGCUGGTT |
| human-XPO1-2# | GGUGCACAAACAGAUCAAATT | UUUGAUCUGUUUGUGCACCTT |
| human-p65-1# | CCCUAUCCCUUUACGUCAUTT | AUGACGUAAAGGGAUAGGGTT |
| human-p65-2# | GAUGAAGACUUCUCCUCCATT | UGGAGGAGAAGUCUUCAUCTT |

**Table S2: The sequences of gene-specific primers used for RT-qPCR and ChIP assay**

| Gene | Forward (5'-3') | Reverse (5'-3') |
|---|---|---|
| Primers for RT-qPCR | | |
| CDC7 | TGGTCTGCAGGTGTCATATTTCT | TCTCAAGTCTTGTGCTGGAACTT |
| CCND1 | CCTCGGTGTCCTACTTCAAATGT | TTCATCTTAGAGGCCACGAACAT |
| CDKN1A | CTGTCACTGTCTTGTACCCTTGT | CCCAGCAGAGGAACCACTACTA |
| DBF4 | CCCTGCAGTCCATTTGATGTAGA | ACTGGAGTTGAATTGAGGTTCCA |
| BCL2 | GGATTGTGGCCTTCTTTGAGTTC | CTTCAGAGACAGCCAGGAGAAAT |
| BCL2L1 | GTGGAAAGCGTAGACAAGGAGAT | GCTGCATTGTTCCCATAGAGTTC |
| MCL1 | TGCTTCGGAAACTGGACATCA | TAGCCACAAAGGCACCAAAAG |
| MYC | CAAGAGGCGAACACACAACG | GTCGTTTCCGCAACAAGTCC |
| XPO1 | AGCAAAGAATGGCTCAAGAAGT | TATTCCTTCGCACTGGTTCCT |
| P65 | ATGTGGAGATCATTGAGCAGC | CCTGGTCCTGTGTAGCCATT |
| GADPH | ACAGTCCATGCCATCACTGCC | GCCTGCTTCACCACCTTCTTG |
| Primers for ChIP assay | | |
| DBF4 promoter for p65 (site A) | CTTCCCAATTCTGTGGCATT | CTTGAGTGGTGGAAGGAAGTG |
| DBF4 promoter for p65 (site B) | TTTTTGGAGAGGCATGTTCC | ACAGCAAGCGGGGTTTTC |
| DBF4 promoter for p65 (site C) | AAAGCCCCTCGCCTTCAG | GCGCAGCCTCAGAGAGTT |

| | | |
|---|---|---|
| XPO1 promoter for p65 (site A) | GCTGGGATTAAAGGCATGAA | CAAAATAAAACGCAATGCAG |
| XPO1 promoter for p65 (site B) | CAAAACATGCGCTGTCAAAC | GCCCCAGACCTCTCTAATCC |
| XPO1 promoter for p65 (site C) | TCTCCGTCACCGAAGAGG | CCTCTTTTCAGCAGCGATTT |

**Table S3: Antibodies used in this work**

| Antibody | Source | Identifier |
|---|---|---|
| anti-DBF4 | Abcam | ab124707 |
| anti-DBF4 | Santa Cruz | sc-293398 |
| anti-CDC7 | Abcam | ab229187 |
| anti-GADPH | CST | 5174 |
| anti-p65 | ABclonal | 5970 |
| anti-Exportin-1 | CST | 46249 |
| anti-Exportin-1 | ABclonal | A19625 |
| anti-Histone H3 | CST | 60932 |
| anti-c-Myc | Santa Cruz | sc-42 |
| anti-STAT3 | CST | 8768 |
| anti-STAT3 | Thermo Scientific | MA1-13042 |
| anti-Phospho-STAT3 (Tyr705) | CST | 9145 |
| anti-Phospho-STAT3 (Ser727) | CST | 49081 |
| anti-JAK1 | ABclonal | A11963 |
| anti-JAK2 | ABclonal | A19629 |
| anti-JAK3 | CST | 8827 |
| anti-Flag tag | CST | 14793 |
| anti-HA tag | ABclonal | AE008 |
| anti-HA tag | Abcam | ab9110 |
| anti-Myc tag | Abcam | ab32 |

| | | |
|---|---|---|
| anti-PD-1 | Abcam | Ab214421 |
| anti-GFP | CST | 2955 |
